# Anti-inflammatory and anti-oxidative electrospun nanofiber membrane promotes diabetic wound healing via macrophage modulation

**DOI:** 10.1186/s12951-024-02385-9

**Published:** 2024-03-16

**Authors:** Jibing He, Shasha Zhou, Jiaxing Wang, Binbin Sun, Dalong Ni, Jinglei Wu, Xiaochun Peng

**Affiliations:** 1 Department of Orthopedics, Shanghai Sixth People’s Hospital, Shanghai Jiao Tong University School of Medicine, Shanghai, 200233 P. R. China; 2 Shanghai Engineering Research Center of Nano-Biomaterials and Regenerative Medicine, College of Biological Science and Medical Engineering, Donghua University, Shanghai, 201620 P. R. China; 3 Department of Orthopaedics, Shanghai Key Laboratory for Prevention and Treatment of Bone and Joint Diseases, Shanghai Institute of Traumatology and Orthopaedics, Ruijin Hospital, Shanghai Jiao Tong University School of Medicine, Shanghai, 200025 P. R. China

**Keywords:** Electrospinning, Nanofiber membrane, Itaconic acid, Anti-inflammatory, Diabetic wound

## Abstract

**Background:**

In the inflammatory milieu of diabetic chronic wounds, macrophages undergo substantial metabolic reprogramming and play a pivotal role in orchestrating immune responses. Itaconic acid, primarily synthesized by inflammatory macrophages as a byproduct in the tricarboxylic acid cycle, has recently gained increasing attention as an immunomodulator. This study aims to assess the immunomodulatory capacity of an itaconic acid derivative, 4-Octyl itaconate (OI), which was covalently conjugated to electrospun nanofibers and investigated through in vitro studies and a full-thickness wound model of diabetic mice.

**Results:**

OI was feasibly conjugated onto chitosan (CS), which was then grafted to electrospun polycaprolactone/gelatin (PG) nanofibers to obtain P/G-CS-OI membranes. The P/G-CS-OI membrane exhibited good mechanical strength, compliance, and biocompatibility. In addition, the sustained OI release endowed the nanofiber membrane with great antioxidative and anti-inflammatory activities as revealed in in vitro and in vivo studies. Specifically, the P/G-CS-OI membrane activated nuclear factor-erythroid-2-related factor 2 (NRF2) by alkylating Kelch-like ECH-associated protein 1 (KEAP1). This antioxidative response modulates macrophage polarization, leading to mitigated inflammatory responses, enhanced angiogenesis, and recovered re-epithelization, finally contributing to improved healing of mouse diabetic wounds.

**Conclusions:**

The P/G-CS-OI nanofiber membrane shows good capacity in macrophage modulation and might be promising for diabetic chronic wound treatment.

**Supplementary Information:**

The online version contains supplementary material available at 10.1186/s12951-024-02385-9.

## Introduction

Diabetes mellitus (DM), a prevalent chronic metabolic disorder, affects an estimated 536 million people globally [1]. Approximately 25% of these patients eventually develop diabetic foot ulcers (DFUs), with an amputation rate of 43.8% and a mortality rate of 51.7% within 6 years post-hospitalization for the ulcer, resulting in an estimated global economic burden of $8.5 billion [2]. Conservative medical interventions, including antibiotics, magnetic thermal therapy, and hyperbaric oxygen chamber therapy, often show unsatisfactory outcomes and are sometimes associated with moderate-to-severe side effects [3, 4]. Therefore, there is an urgent need for new therapies to address chronic diabetic wounds.

The process of wound healing includes three overlapping stages: inflammation, proliferation, and remodeling [5]. Macrophages, as the most active non-specific immune cells throughout the entire process, play a double-edged sword role [6]. On the one hand, the activation of pathogen-associated molecular patterns (PAMPs) and damage-associated molecular patterns (DAMPs) biases macrophage polarization toward M1 phenotype in chronic inflammatory milieu, leading to the secretion of pro-inflammatory mediators [7]. On the other hand, an increased transformation of M2 phenotype macrophages in the latter stages secretes pro-healing chemokines and growth factors, which foster the recruitment of endothelial progenitor cells that are essential for neovascularization and ensure sufficient blood flow to the wound site [8]. Generally, in normal wound healing, M1 macrophages dominate from day 1 to day 3, followed by a shift towards M2 macrophages, peaking at around day 7 [9]. However, in the typical hyperglycemic microenvironment of diabetic conditions, the phenotypic shift of macrophages is dysregulated. Regardless of the time point after the initial injury, the wound microenvironment is consistently dominated by M1 macrophages with abnormally high release of inflammatory cytokines and chemokines [10]. These alterations lead to the recruitment and infiltration of immune cells into the wound tissues. Furthermore, the excessive accumulation of immune cells greatly increases the local oxygen consumption in chronic wounds, impairing the processes of angiogenesis and epithelial regeneration. This further delays the healing of diabetic wounds, triggering a vicious cycle [11, 12]. Thus, strategies that modulate the physiological roles of macrophages pose significant implications for diabetic wounds.

Intermediates in macrophage glycolysis and the tricarboxylic acid cycle (TAC), such as lactic acid and citric acid, are increasingly recognized as key elements affecting macrophage function and phenotype [13, 14]. Previous studies have demonstrated that activated M1 macrophages exhibit elevated expression of immune-responsive gene 1 (IRG1), which encodes aconitate decarboxylase and further catalyzes the conversion of the tricarboxylic acid cycle intermediate cis-aconitate into itaconic acid [15]. Structurally akin to methylene, itaconic acid modulates immune responses through the competitive inhibition and alkylation of target proteins [16]. Specifically, itaconic acid impedes the activity of succinate dehydrogenase in macrophages, and reverses the mitochondrial electron transport chain, thereby reducing inflammation by diminishing reactive oxygen species (ROS) production and altering macrophage metabolism [17]. 4-Octyl itaconate (OI), a derivative of itaconic acid, is more permeable to cell membranes than its parent compound [18]. Previous studies have demonstrated that OI can enter macrophages and be converted into itaconic acid to regulate mitochondrial metabolism [19]. Therefore, in this study, we selected OI as a targeted therapeutic agent for modulating macrophage activity in the chronic inflammatory microenvironment of diabetic wounds.

Nowadays, many biomaterials with multifaceted functionalities, including anti-inflammatory, reparative, and antibacterial properties, have been developed for resolving chronic inflammation and facilitating diabetic wound healing [20, 21]. However, despite these promising functionalities, how to balance the biological and mechanical properties of these materials to achieve optimal in vivo effectiveness remains a critical challenge yet to be resolved. Chitosan (CS), a linear, semi-crystalline natural polysaccharide derived from chitin, can act as a biocompatible, biodegradable biological scaffold with antioxidant and antibacterial properties [22]. Electrospun nanofiber membranes are featured by their biomimetic structure, high porosity, extensive surface area, softness, and compliance and have been extensively investigated for wound dressing applications [23]. Especially, CS-coated electrospun nanofiber membranes show great antibacterial and antioxidative activities and are attractive for wound treatment [24, 25].

**Scheme 1:**
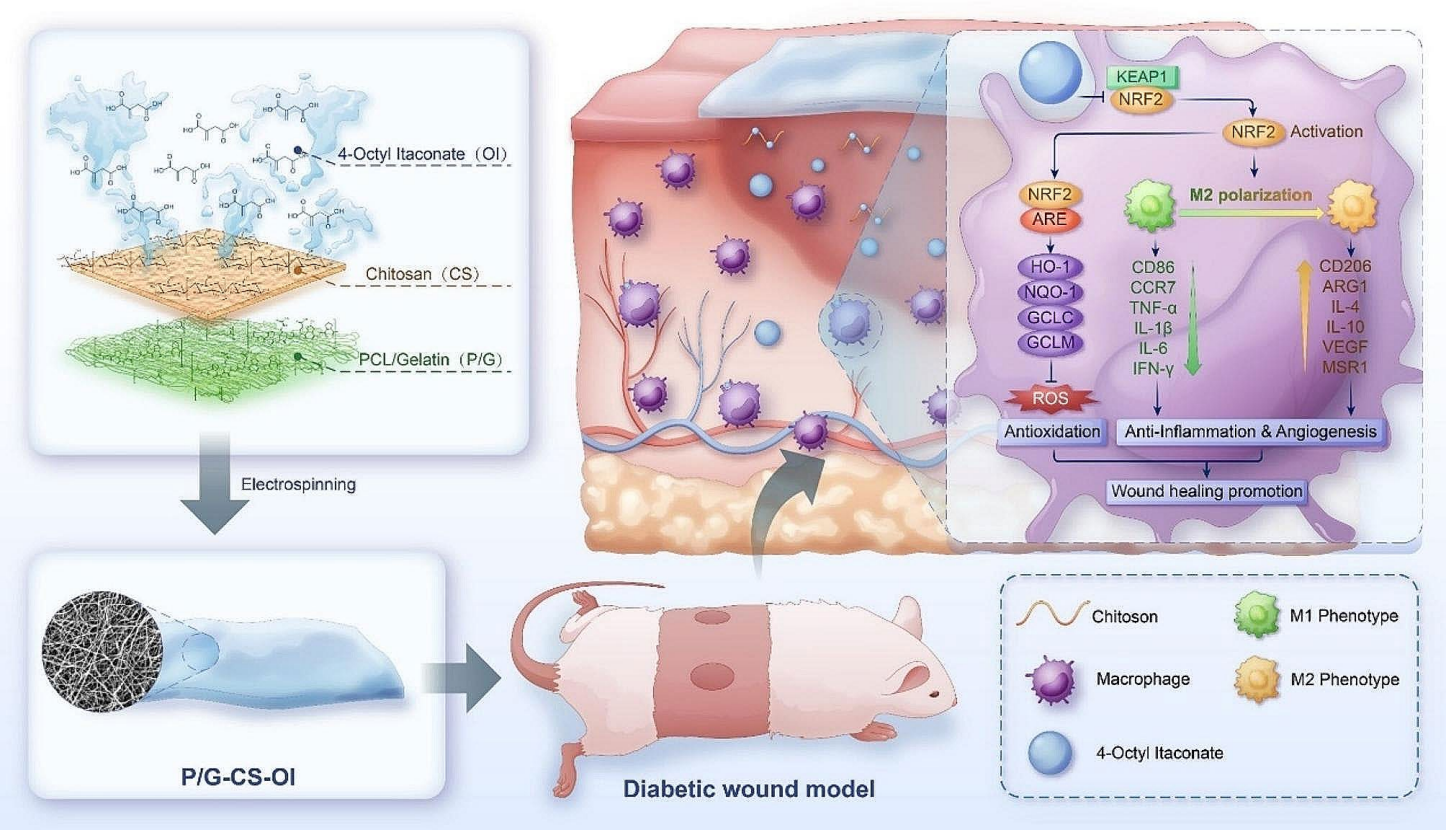
Schematic illustration of a Chitosan-4-Octyl itaconate grafted nanofiber membrane (P/G-CS-OI), which can promote diabetic wound healing via macrophage modulation

To promote diabetic wound healing, rescuing macrophages from the abnormal inflammatory environment of diabetic wounds could be an effective approach. We herein reported a strategy to prepare OI-grafted nanofiber membrane by electrospinning and assessed its anti-inflammatory capacity in vitro and biological performance in treating diabetic wounds in a mouse model (Scheme 1). We hypothesized that the covalently conjugated OI could show sustained release from nanofibers and have modulatory effects on macrophage polarization, which can be harnessed to treat diabetic wounds. To this end, OI was conjugated to CS to obtain CS-OI conjugate, which was then covalently grafted to electrospun polycaprolactone (PCL)/gelatin nanofibers to obtain PCL/gelatin-CS-IO (P/G-CS-OI) membrane. Physicochemical properties of the P/G-CS-OI membrane were characterized and its anti-inflammatory, antibacterial, and antioxidative activities, as well as biocompatibility and bioactivity, were assessed. Finally, the P/G-CS-OI membrane was used as dressings in a diabetic mouse model to assess its biological performance for curing chronic wounds.

## Materials and methods

### Materials

Polycaprolactone (PCL, Mn = 80 kDa) and type A gelatin (300 g Bloom) were obtained from Sigma Aldrich (St. Louis, Missouri, USA). Chitosan (CS, 90% deacetylated powder, Mw = 200 kDa), 4-octenyl itaconate (OI), and 2-(N-morpholino) ethanesulfonic acid (MES) were purchased from Macklin Biochemical Co., Ltd. (Shanghai, China). Glacial acetic acid and 2,2,2-trifluoroethanol (TFE) were obtained from Meryer Chemical Technology Co., Ltd. (Shanghai, China). Adhesive bandage was obtained from Hainuo Medical Technologies Co., Ltd. (Qingdao, China). 1-ethyl-3-(3-dimethylaminopropyl) carbodiimide (EDC) was purchased from Sinopharm Chemical Reagent Co., Ltd. (Shanghai, China). N-hydroxysuccinimide (NHS) was obtained from Adamas-beta (Shanghai, China). LB Broth and LB Broth Agar were purchased from Sangon Biotech (Shanghai, China).

### Electrospinning of P/G membranes

PCL and type A gelatin were dissolved in TFE to achieve a total polymer concentration of 10% (w/v). The ratio of PCL to gelatin was 8:2, and the mixture was stirred at room temperature to ensure homogeneity. To enhance the miscibility between PCL and gelatin, a small quantity (0.2%, v/v) of glacial acetic acid was added to the solution. The electrospinning process was conducted by feeding this solution through a 20 G needle at a consistent rate of 1.5 mL/h under a charge of 15 kV voltage to generate nanofibers in an atmospheric environment with a relative humidity of ∼ 50–60%. The as-spun nanofibers were collected on a slowly rotating mandrel (60 mm diameter, rotating at 120 rpm) to obtain PCL/gelatin (P/G) membranes.

### Preparation of P/G-CS-OI membranes

The synthesis of the CS-OI conjugate was achieved using EDC/NHS carbodiimide chemistry, which facilitates the formation of amide bonds between the amino groups of CS and the carboxyl groups of OI. CS was dissolved in 0.1 M dilute hydrochloric acid to a concentration of 1% (w/v). Subsequently, OI, EDC (1-ethyl-3-(3-dimethylaminopropyl) carbodiimide), and NHS (N-hydroxysuccinimide) were added in succession, ensuring a final molar ratio of CS:OI:EDC:NHS at 50:1:10:5. The reaction was maintained at room temperature in a dark environment for 24 h. Then the reaction was air-dried, washed with ethanol to remove any unreacted residues, and air-dried again to obtain the CS-OI conjugate.

For the functionalization, membranes were immersed in a CS-OI aqueous solution (1 mg/mL ) at 37 °C and agitated for 24 h. Post-infusion with the CS-OI conjugate, the membranes were soaked in MES (2-(N-morpholino) ethanesulfonic acid) buffer to facilitate the EDC/NHS crosslinking process at room temperature for 24 h. To remove any unreacted EDC and NHS, samples were washed extensively with deionized water, undergoing 6 cycles of 30 min each. Finally, electrospun nanofiber membranes were vacuum-dried in an oven to remove residual solvent for further studies.

### Characterization of P/G-CS-OI membranes

The morphology of electrospun membranes was observed by scanning electron microscope (SEM, Phenom, XL, Netherlands). Fiber diameters of P/G-CS-OI membranes were measured from SEM micrographs by Image J. For each electrospun membrane, at least 100 fibers were randomly selected from 3 independent samples for measurement. An fourier transform infrared reflection (FTIR) spectrometer (Nicolet iS 10, Thermo Fisher Scientific, USA) was used to identify the chemical structures of electrospun nanofiber membranes. The FTIR spectra were obtained by recording 32 scans between 4000 and 400 cm^− 1^ with a resolution of 0.5 cm^− 1^. The X-ray Diffraction (XRD) patterns of CS-OI were determined by an X-ray diffractometer (D8 Advance, Bruker, Germany), detecting the crystalline phase of samples at 2θ between 5° and 60°.

We employed the SL200A contact angle analyzer (Solon Tech., Shanghai, China) to obtain the water contact angle (WCA) of nanofiber membranes. A water droplet (5 μL) was placed on the surface of nanofiber membranes and the dynamic water contact angle was recorded. The contact angle was measured by using Image J (*n* = 3). The mechanical properties of electrospun membranes were evaluated using an uniaxial tensile test as described in our prior publication [26]. Membranes were tailored into rectangular samples (10 × 40 mm) and underwent incubation in phosphate-buffered saline (PBS) at a temperature of 37 ℃ for 24 h. Afterward, samples were positioned within the grips of a uniaxial testing machine (Instron 5567, Norwood, MA) equipped with a 50 N load cell and tested until failure at a crosshead speed of 10 mm/min. The ultimate tensile strength (UTS) was defined as the maximum load before failure and Young’s modulus was calculated as the slope of the stress-strain curve’s initial 5% linear section (*n* = 3).

The water absorption and moisturizing rate of electrospun membranes were measured by weighing. To determine the water absorption rate, electrospun nanofiber membranes were tailored into 1 × 1 cm squares and weighed (W_0_), then immersed in deionized water at room temperature, wiping away the surface water carefully using filter paper and weighing again (W_t_) (*n* = 3). The water absorption rate of membranes was calculated by the following equation:

$$water\,absorption\,rate\,\left( \% \right) = ({W_t} - {W_0})/{W_0} \times 100\%$$

To determine the moisturizing rate, electrospun nanofiber membranes were tailored into 1 cm ×1 cm squares and weighed (W_0_) and incubated in deionized water for 20 min at room temperature, and weighed (W_1_). Membranes were placed at room temperature environment and weighed (W_2_) at predesignated time points (*n* = 3). The moisturizing rate of membranes was calculated by the following equation:

$${\rm{moisturizing}}\,{\rm{rate}}\,\left( \% \right)\, = \,({W_2} - {W_0})/({W_1} - {W_0}) \times 100\%$$

In vitro degradation and drug release were conducted by immersing electrospun membranes in PBS under a shaker incubator at 37 °C. To determine the in vitro degradation, electrospun nanofiber membranes were tailored into 1 cm ×1 cm squares and weighed (W_0_). Incubated samples were retrieved, rinsed with deionized water, lyophilized, and weighed (W_w_) at predesignated time points (*n* = 3). The remaining mass was determined according to the following equation:

$$Percentage\,of\,remained\,mass\,\left( \% \right)\, = \,\frac{{{W_w}}}{{{W_0}}} \times 100\%$$

The drug release of OI from the P/G-CS-OI membrane was assessed through ultraviolet (UV)-visible spectrophotometry. UV analysis was conducted on the incubated solution (1 mL) at specified time intervals, and an equivalent volume of fresh PBS was substituted. The concentration of OI released from P/G-CS-OI membranes was determined by comparing it to the standard curve based on known OI concentrations (*n* = 6).

### Antibacterial assessment

The antibacterial activity of the P/G-CS-OI membranes was evaluated against two commonly found bacterial species. More specifically, *E. coli* (ATCC 25,922) and *S. aureus* (ATCC 25,923) were cultured in Luria broth under shaking conditions for 24 h at a temperature of 37 ℃. Subsequently, each sample was inoculated with 200 μL of bacterial inoculum solution (10^8^ CFU/mL) and then incubated at 37 ℃ for 24 h. The bacterial solution was then diluted and cultured with 100 μL of diluent on LB Agar for 12 h. Bacterial colonies were imaged using an automated colony counter (Shineso Science & Technology Co., Ltd, Hangzhou). Colonies were counted using Image J to calculate the normalized survival rate (*n* = 3).

### Cell culture

The RAW 264.7 macrophages and human immortalized keratinocytes (HaCaT) were maintained in Dulbecco’s Modified Eagle Medium (DMEM), supplemented with 10% fetal bovine serum (FBS) and 1% penicillin/streptomycin (P/S). Cells were incubated at 37 °C in a humidified atmosphere containing 5% CO_2_, with the culture medium being refreshed every two days to maintain optimal cell growth and viability. Before cell seeding, electrospun nanofiber membranes were punched using a sterile punch to match the dimensions of the wells in standard cell culture plates. Samples were subjected to UV sterilization to ensure aseptic conditions.

### Cytocompatibility assessments

RAW 264.7 macrophages and HaCaTs were revived and cultured on the surface of membranes in 48-well plates at a density of 20,000 cells per well. The cultures were maintained at 37 °C, with the culture medium changing every other day.

Cell viability and proliferation on the electrospun nanofiber membranes were assessed at 1, 4, and 7 days post-seeding. Before the test, the culture medium was aspirated, and the cells were washed thrice with PBS. Then, DMEM medium containing Cell Counting Kit-8 (CCK8; Biosharp) was added to 96-well plates and incubated with RAW 264.7 cells or HaCaTs for 2 h. The absorbance was measured at 450 nm with a microplate reader (Multiskan MK3, Thermo, USA) to assess cell viability (*n* = 9). Cells were stained with live/dead staining reagents according to the manufacturers instructions. Images were acquired on an inverted fluorescence microscope (DMi 8, Leica, Germany) to count the numbers of live and dead cells (*n* = 3). Cell survival rate was calculated using the formula: cell survival rate (%) = (Viable cells/Total cells) × 100%.

In addition to these quantitative assays, cell adhesion and morphology on the nanofiber membranes were visually analyzed. At day 7, the cells cultured on the membranes were fixed with 4% paraformaldehyde, sequentially dehydrated in graded ethanol solutions, and air-dried at room temperature. The samples were then sputter-coated with gold to enhance conductivity and imaged using a scanning electron microscope (Phenom, XL, Netherlands).

### Flow cytometry

The electrospun nanofiber membranes were first placed in 6-well plates, and RAW264.7 cells were seeded on their surfaces at a density of 2 × 10^5^ cells per well. Following the incubation, the culture medium was discarded, and the cells were washed three times with PBS to remove debris and non-adherent cells. Lipopolysaccharide (LPS, TargetMol) was then added to the culture to activate macrophages. After another 24 h, cells were collected for analysis.

The cell pellets were resuspended in 100 μL of PBS containing 1.25 μg of PE-conjugated anti-mouse CD86 antibody and 0.25 μg of FITC-conjugated anti-mouse CD206 antibody (Biolegend). This step was performed on ice for 30 min to preserve cell viability and prevent nonspecific antibody binding. The expression of CD86 and CD206 was subsequently analyzed using the CytoFLEX flow cytometry system (Beckman Coulter). Additionally, the intracellular ROS level was detected using 2,7dichlorodihydrofluorescein diacetate (DCFH-DA; Yeasen), following the same procedure as described above (*n* = 3).

### Scratch assay

As mentioned above, the electrospinning nanofiber membrane was placed on a 6-well plate, and seed RAW264.7 cells on its surface at a density of 2 × 10^5^ cells/well. After the cells have fully adhered, add LPS to activate the macrophages. After 6 h, wash three times with PBS, incubate with complete DMEM medium for 24 h, then remove the medium and mix it 1:1 with fresh complete DMEM medium to obtain macrophage-conditioned medium. HaCaTs were seeded into a new 6-well plate at a density of 1 × 10^5^ cells per well. Upon reaching approximately 90% confluence, a scratch was carefully made through the cell monolayer to simulate a wound. Afterwards, macrophage-conditioned medium was added to the wells. To monitor and quantify the process of wound closure, three random fields of view along the scratch were selected in each well. These areas were photographed using an optical microscope (IX53, Olympus, Japan) at 0, 24, and 48 h post-scratch. Scratch width were measured from micrographs by using Image J (*n* = 6). The healing rate (%) was determined according to the following equation:

$${\rm{Healing}}\,{\rm{rate}}\,\left( \% \right)\, = \left( {1 - \frac{{new\,scratch\,width}}{{original\,scratch\,width}}} \right) \times 100\%$$

### Real‑time polymerase chain reaction (RT‑PCR)

Cell culture of RAW264.7 and HaCaTs were as aforementioned for RNA extraction. Total RNA was extracted from the samples using the EZ-press RNA Purification Kit (EZBioscience, Roseville, MN, USA). Following extraction, the RNA was reverse transcribed into complementary DNA (cDNA) utilizing the Colour Reverse Transcription Kit (EZBioscience). Quantitative RT-PCR was then performed using the 2 × Color SYBR Green qPCR Master Mix (EZBioscience). The relative expression levels of the target genes were calculated employing the 2^−ΔΔCt^ method (*n* = 3). Primer sequences are shown in Table S1 (Additional file), and GAPDH was used as internal reference gene.

### Hemocompatibility assessments

Fresh blood was collected from healthy mice and centrifuged at 1000 rpm for 10 min to isolate pure red blood cells (RBCs). The RBCs were washed three times and subsequently diluted in PBS to achieve a final concentration of 2%. For the assay, electrospun nanofiber membranes with a diameter of 8 mm were introduced into 250 μL of the RBC suspension and incubated at 37 °C for 1 h. As controls, saline and deionized water were employed as the negative and positive controls, respectively, and mixed with the RBC suspension under identical conditions. Following the incubation period, all samples underwent centrifugation at 1000 rpm for 5 min.

The supernatant of each sample was then analyzed spectrophotometrically to measure the absorbance of released hemoglobin at 540 nm with a microplate reader (Multiskan MK3, Thermo, USA). The hemolysis ratio, indicative of the degree of damage to the RBCs, was calculated using the following formula: Hemolysis ratio (%) = [(Abs - Absn)/(Absp - Absn)] × 100%, where Abs represents the absorbance of the sample, Absp the absorbance of the positive control, and Absn the absorbance of the negative control (*n* = 3).

### Subcutaneous implantation

Electrospun membranes were punched into 8 mm discs and sterilized by UV (12 h for each side) and were subcutaneously implanted in seven-week-old female rats (weighing approximately 280–320 g, Shanghai Slack Laboratory Animal Limited Liability Company, Shanghai). The rats were anesthetized by intraperitoneal injection of chloral hydrate (10%), and pockets were created by blunt dissection of the fascia between the skin and muscle. Electrospun membranes were embedded in the four pockets of the same rat and closed with sutures (*n* = 4 for each group at each time point). Six animals were used for subcutaneous implantation. After 7 and 28 days, the entire subcutaneous tissue containing electrospun nanofiber membranes was excised, and fixed with 4% paraformaldehyde overnight. Paraffin-embedded tissues and sectioned into 5 μm sections for hematoxylin and eosin (H&E) and Masson’s trichrome staining. Images of histological slides were taken using an optical microscope (L608-3M2000, AOSVI, Shenzhen). Cell infiltration depth and fibrous capsule thickness were measured from H&E and Masson’s trichrome staining images by Image J, respectively. For each group at each time point, nine measurements of independent areas from histological images were averaged.

### Streptozotocin (STZ)‑induced diabetic mice

The animal study protocols were rigorously designed and executed in compliance with the ethical guidelines set forth by the Institutional Animal Care and Use Committee (IACUC) of Shanghai Sixth People’s Hospital Affiliated with the Shanghai Jiao Tong University School of Medicine. A cohort of 36 male ICR mice, aged between 6 and 8 weeks, was utilized for the establishment of a diabetic model. The mice were housed under controlled environmental conditions, maintained at 22 ± 2 ℃, with three animals per cage to ensure adequate space and social interaction. The diabetic state in the mice was induced by 150 mg/kg of STZ (≥ 98% purity; Beyotime). To enhance the effectiveness of STZ, mice were subjected to a 24 h fasting before the administration of STZ. Subsequently, the blood glucose levels of mice were monitored regularly by sampling from the tail vein 3 days post-injection. A continuous blood glucose level greater than or equal to 16.7 mM was considered the successful establishment of diabetes.

### Wound healing evaluation

After anesthesia, the dorsal fur of the mice was carefully removed using an electric animal razor, and the surgical area was disinfected with 75% alcohol. Mice were subsequently divided into three distinct groups: Control, P/G-CS, and P/G-CS-OI, with each group comprising 12 animals. In each mouse, a full-thickness skin wound of 8 mm diameter was created on the back using a sterile biopsy punch. The wounds were then covered with the respective electrospun nanofiber membranes, wrapped by adhesive bandage and secured with sutures. The progression of wound healing was documented at 0, 3, 7, 10, and 14 days post-wounding by capturing digital photographs of the wounds. In addition, the wound tissues of each group were randomly selected and stored in liquid nitrogen at day 7 for subsequent RT-PCR and ELISA detection.

### RT-PCR and ELISA assays

After tissue homogenization and digestion, macrophages were separated from the digestive tissue by flow sorting. The subsequent RNA extraction, RT-PCR and data analysis methods were referred to the above methods, and GAPDH was used as the internal reference gene. The remaining homogenates were then centrifuged at 12,000 g for 5 min, and the supernatants were collected. The expression levels of various cytokines were quantitatively assessed using an ELISA kit (Servicebio, Wuhan, China), following the manufacturer’s instructions (*n* = 3).

### Histological, immunofluorescence, and immunohistochemical analyses

Healing tissues of animals 3, 7, 10, and 14 days post-surgery were harvested and subjected to a series of histological preparations. Samples were then stained using H&E (Solarbio) and Masson’s trichrome (Solarbio). Immunofluorescence (IF) and immunohistochemical (IHC) staining were performed to evaluate the macrophage phenotype and extent of tissue repair in the injured skin tissues. The sample sections were incubated with primary and secondary antibodies respectively. Specific antibodies including CD31 (1:4000; Proteintech), CD86 (1:200; Abways), CD206 (1:300; Proteintech), α-SMA (1:300; Proteintech), Keratin 10 (K10) (1:100; Abways), Keratin 14 (K14) (1:100; Abways), tumor necrosis factor (TNF)-α (1:50; Abways), interleukin (IL)-10 (1:50; Proteintech), vascular endothelial growth factor (VEGF) (1:50; Abways), NRF2 (1:200; Proteintech). And Goat Anti-Mouse IgG (H + L) Cy3 (1:200; Abways), Goat Anti-Rabbit IgG (H + L) Alexa Fluor 488 (1:200; Abways), HRP-conjugated Goat Anti-Rabbit IgG Heavy Chain (1:5000; Abclonal) were used to label the target cells and proteins. The stained sections were examined under a Ci-S microscope (Nikon) for morphological and quantitative analysis.

### Statistical analysis

Data were expressed as mean ± standard deviation (SD). Statistical analysis was performed using GraphPad Prism. One-way or two-way analysis of variance (ANOVA) with Tukey’s post hoc multiple comparisons were used to analyze significance, where appropriate. A *p*-value of less than 0.05 was considered statistically significant.

## Results and discussion

### Physicochemical properties of electrospun membranes

**Fig. 1:**
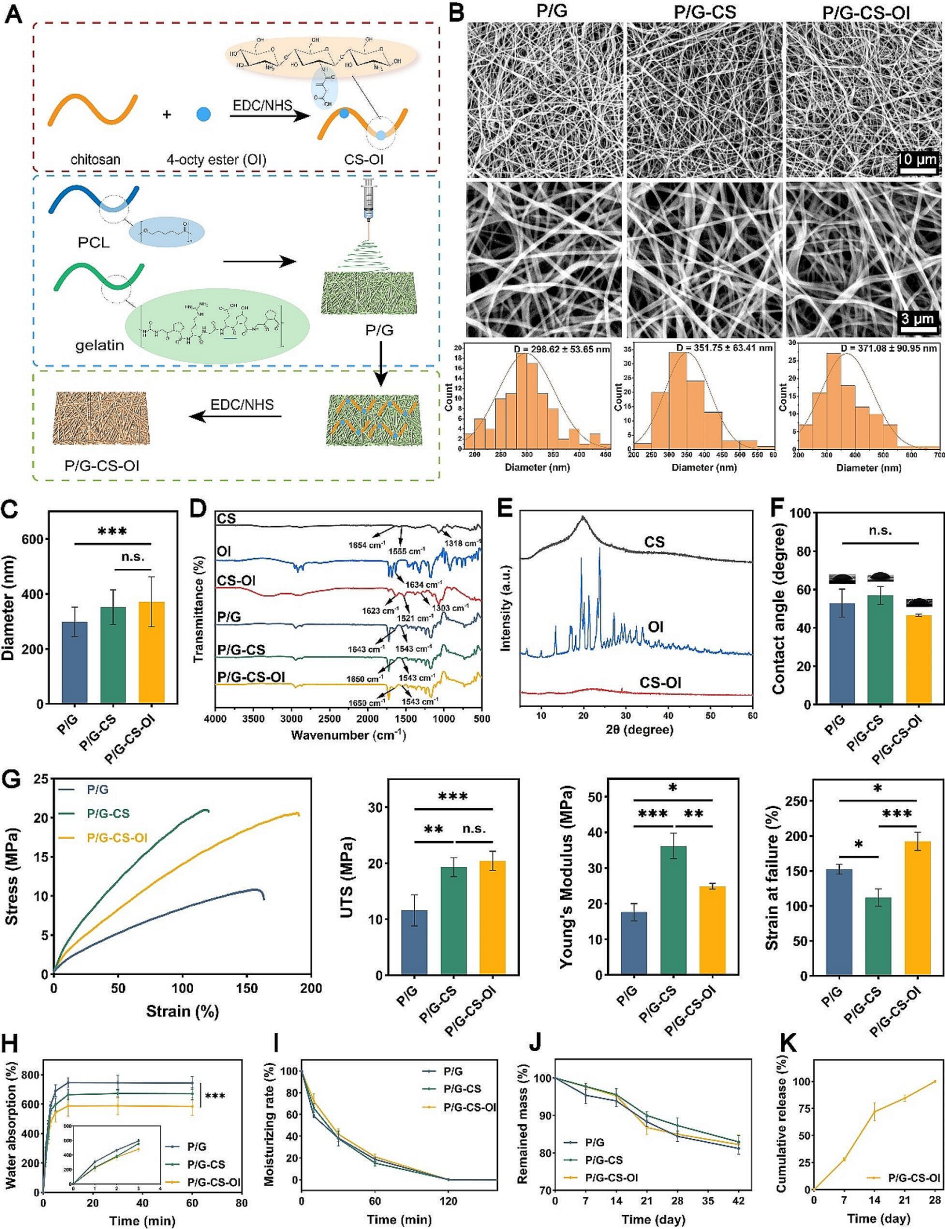
Preparation and physiochemical properties of electrospun nanofiber membranes. Schematic of electrospun nanofiber membrane preparation (**A**). SEM images of electrospun nanofiber membranes (**B**) with nanofiber diameter distribution (**C**). FTIR spectra, (**D**) and XRD patterns (**E**), water contact angle (**F**), mechanical properties (**G**), water absorption rate (**H**), moisturizing rate (**I**), in vitro degradation (**J**), and drug release profile (**K**) of electrospun nanofiber membranes. *p < 0.05, **p < 0.01, and ***p < 0.001, and n.s., not significant

The fabrication and structural characterization of surface-modified nanofiber membranes P/G-CS-OI are detailed in Fig. 1A. SEM images reveal a dense arrangement of porous, randomly oriented nanofibers (Fig. 1B). The average diameters of P/G, P/G-CS, and P/G-CS-OI membranes were 299 ± 54 nm, 352 ± 63 nm, and 371 ± 91 nm, respectively. Covalent grafting of CS and CS-OI to P/G nanofibers led to increased fiber diameter (Fig. 1C) but did not alter the randomly oriented nanofiber structure.

FTIR was employed to analyze the chemical composition of the CS-OI conjugate and electrospun nanofiber membranes (Fig. 1D). CS exhibited characteristic absorption bands of functional groups: amide I (C = O stretching) at 1654 cm^− 1^, amide II (N-H bending) at 1555 cm^− 1^, and amide III (C-N stretching) at 1318 cm^− 1^ [27]. In the OI spectra, the prominent band at 1721 cm^− 1^ corresponded to the C = O stretching of carboxyl groups. For CS-OI conjugate, the band intensity increased at 1643 cm^− 1^ and 1521 cm^− 1^, indicating the formation of amide I and amide II bonds between the carboxyl group of OI and the amino group of the CS chain through amide bonds. In addition, the characteristic absorption band of the OI monomer at 1727 cm^− 1^ was also observed in the CS-OI conjugate, further proving the existence of the OI component in the CS-OI conjugate. Therefore, these results demonstrate that the grafting reaction occurs in the carboxyl group of OI and the amino group of CS through the amide bond. For P/G electrospun membranes, the characteristic peaks of amide I and amide II at 1643 cm^− 1^ and 1543 cm^− 1^, respectively, confirmed the presence of gelatin within the electrospun membranes. For P/G-CS and P/G-CS-OI membranes, the characteristic peak of the amide I band shifts toward a higher wavenumber (from 1643 to 1650 cm^− 1^), and such shift in peak positions implies weakening or lengthening of bonds, confirming the existence of an interaction of the OI with the CS [28, 29].

XRD patterns (Fig. 1E) demonstrate that CS had a broad peak at 2θ = 19.9 showing its high crystallinity, aligning with previous reports [30, 31]. The high crystallinity of chitosan is generally caused by inter- and intra-molecular hydrogen bonds [32]. OI displayed multiple narrow and sharp diffraction peaks that indicate its characteristic of crystalline materials. The CS-OI conjugate exhibited a smooth diffraction pattern, indicating reduced crystallinity and the consequent increase of the amorphous phase, confirming the successful conjugation of OI onto CS. Lower crystallinity would lead to the increase of solubility in water, CS can only be soluble in acid conditions (pKa ∼ 5.5) before reaction and CS-OI were easily soluble in water. This was probably due to that OI hinders the inter- and intra-molecular hydrogen bonds network of chitosan molecules [33, 34]. CS-OI showed water-solubility facilitating subsequent P/G-CS-OI membrane preparation in our study.

Hydrophilicity is a critical property influencing material-tissue contact of wound dressings. The electrospun P/G nanofiber membrane had a water contact angle of 53 ± 7°, indicating its hydrophilic nature (Fig. 1F). This is due to the presence of gelatin that greatly reduces the hydrophobicity of PCL. The introduction of After grafting with CS and CS-OI, the obtained nanofiber membranes had contact angles of 57 ± 5° and 47 ± 1°, respectively, which were not significant from the P/G nanofiber membrane (*p* > 0.05). This result suggests that the overall hydrophilicity is not significantly impacted by the addition of CS or CS-OI.

To guarantee a close and comfortable interaction with the wound, as well as to regulate the tension exerted on it, the mechanical characteristics of the wound dressing should resemble those of human skin. As depicted in Fig. 1G, the UTS for P/G, P/G-CS, and P/G-CS-OI membranes were measured at 12 ± 3 MPa, 19 ± 2 MPa, and 20 ± 2 MPa, respectively, highlighting a marked increase in strength with the conjugation of CS and CS-OI. Moreover, P/G-CS had the highest Young’s modulus (36 ± 4 MPa), surpassing both P/G and P/G-CS-OI (18 ± 2 MPa and 25 ± 1 MPa, respectively). The strains at failure were 153 ± 7% for P/G, 112 ± 13% for P/G-CS, and 192 ± 13% for P/G-CS-OI, P/G-CS-OI membranes exhibited the greatest strains at failure. Human skin typically exhibits a tensile modulus ranging from 15 to 150 MPa, an ultimate tensile stress between 1 and 32 MPa, and an ultimate tensile strain from 35 to 115% [35]. These results indicate that our nanofiber membranes could provide suitable tensile properties for wound dressing.

The water absorption and retention capabilities of nanofiber membranes are critical for their efficacy as wound dressings, particularly in absorbing tissue exudates. The water absorption rates of the nanofiber membranes were evaluated over 60 min, as shown in Fig. 1J. The rates were determined to be 743 ± 42%, 670 ± 40%, and 584 ± 62% for P/G, P/G-CS, and P/G-CS-OI membranes, respectively, with all three types reaching maximum absorption within the first 15 min. In terms of moisture retention, as indicated in Fig. 1H, the rates were 19 ± 2%, 15 ± 3%, and 21 ± 3% for P/G, P/G-CS, and P/G-CS-OI membranes, respectively. These results show that our nanofiber membrane can absorb the solution quickly, reach the maximum absorption ratio in a short time, and remain stable. As a wound dressing, it can maintain a moist environment around the wound, which is favorable for wound healing [36].

In vitro degradation of nanofiber membranes was evaluated in PBS solution at 37 °C for up to 42 days (Fig. 1J). The remaining mass of P/G-CS and P/G-CS-OI samples was around 95% after 2 weeks, decreasing to 82% after 6 weeks. For P/G samples, the remaining mass was 93% in the second week, decreasing to 80%, suggesting a slightly faster degradation rate compared to P/G-CS and P/G-CS-OI membranes. The nanofiber membrane exhibits slow degradation characteristics, which fulfill the requirements of wound dressings. This feature effectively prevents the negative impact of degradation products on the wound. Additionally, it aids in extending the lifespan of the dressing during the wound-healing process, reducing the need for frequent dressing replacements. As a result, it minimizes the secondary trauma caused by dressing changes [37].

The slow degradation of the membrane favors the long-term release of the active ingredient, and we evaluated the cumulative release of OI from the P/G-CS-OI membranes for up to 28 days (Fig. 1K). Within the first 2 weeks, P/G-CS-OI membranes released 75% of OI and exhibited a relatively slow release of OI for a period of up to 28 days. In our study, conjugating small molecule OI to polymeric carries CS has been applied to increase the bioavailability of OI, and the drug delivery system can be further stabilized by crosslinking CS-OI conjugates to nanofibers. In the drug release experiments, P/G-CS-OI membranes achieved long-term release effects, which help to play the biological role of OI for a long time to help skin repair.

The impaired immune defense system induced by diabetes can easily lead to recurrent bacterial infections in chronic hard-to-heal wounds, and the increasing exudate provides a breeding ground for bacterial growth [38]. The antibacterial efficacy of the nanofiber membranes was assessed against common wound pathogens, *E.coli* (Gram-negative) and *S.aureus* (Gram-positive) (Fig. S1, Additional file). The control and P/G groups showed more bacterial colonies, while the P/G-CS and P/G-CS-OI groups had significantly fewer bacterial colonies. The normalized survival of *E. coli* co-cultured with P/G-CS and P/G-CS-OI membranes was 60 ± 7% and 71 ± 2%, respectively, and *S. aureus* was 74 ± 9% and 63 ± 3%, respectively. It should be admitted that the P/G-CS-OI membrane is not as powerful as nanofiber membranes incorporated with other antibacterial agents such as magnesium oxide, zinc oxide, and silver in the literature [39–41]. This is related to the antibacterial mechanism of CS. Despite the antibacterial effect of OI [42], we believe that the antibacterial effects of the P/G-CS-OI membrane are primarily owing to CS. This is because CS is well-known for its good antibacterial properties [43], and the weight of introduced CS is far more than OI in the P/G-CS-OI membrane. In the acidic microenvironment of wounds, its amino group is positively charged, and it can bind with the negative charge on the surface of the bacterial cell membrane, which alters the permeability of the cell membrane and leads to metabolic disorders of the bacterium, to achieve the antibacterial effect [44]. In the CS-OI conjugate, a portion of the amino group of CS was covalently coupled with the carboxyl group of OI, and for the P/G-CS-OI membranes, the amino group of CS was further substituted, which compromises the antibacterial properties of P/G-CS-OI membranes. However, it is might suitable for the prevention of bacterial infection in wound care.

### Biocompatibility of electrospun membranes

**Fig. 2:**
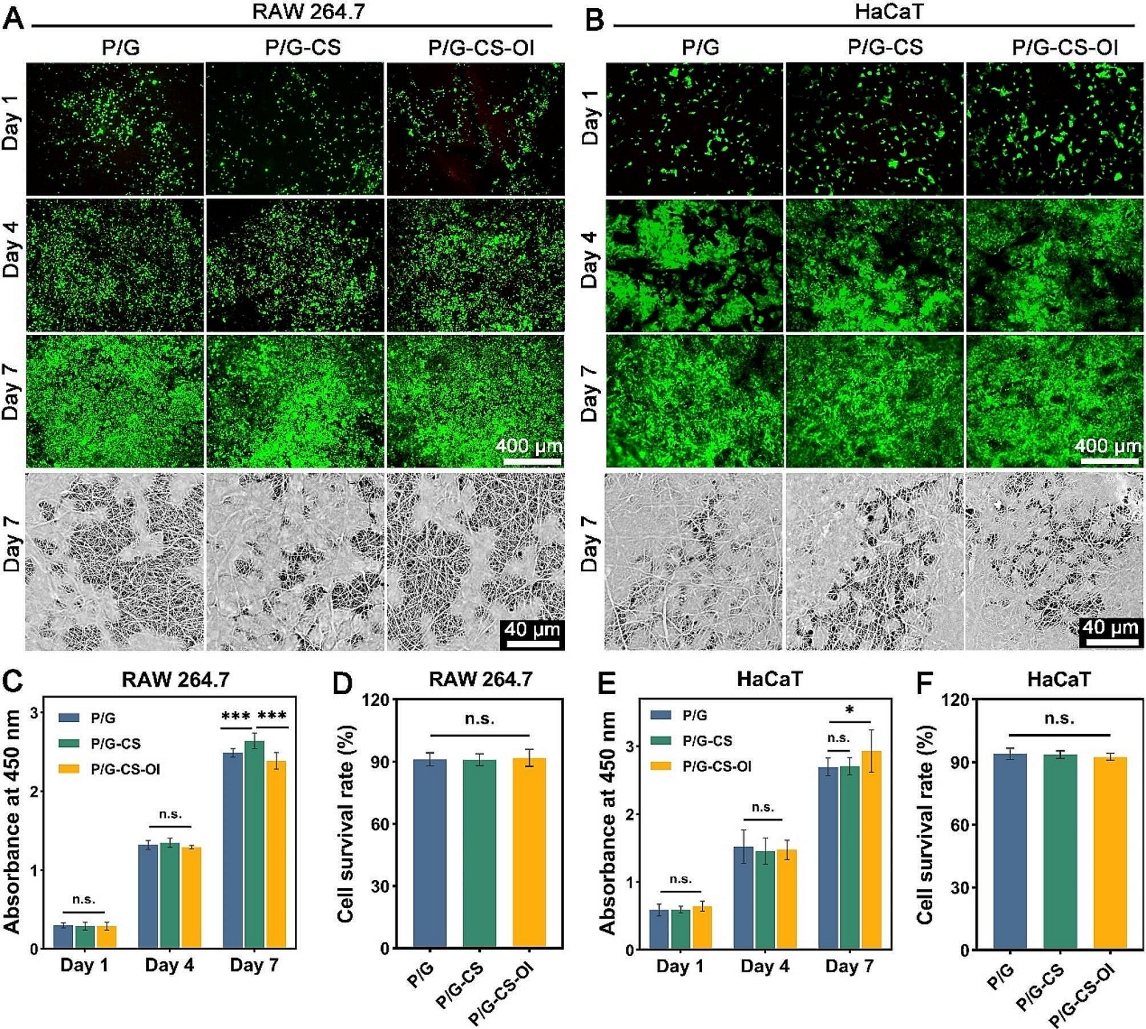
Cytocompatibility assessments. Live/dead staining and SEM images of RAW 264.7 macrophages (**A**) and HaCaTs (**B**) on electrospun nanofiber membranes. Cell proliferation rates (**C** and **E**) were assessed by CCK-8 assay. Cell survival rates (**D** and **F**) were determined by live/dead staining at day 1.*p < 0.05, ***p < 0.001, and n.s., not significant

The cytocompatibility of electrospun membranes was evaluated with RAW 264.7 and HaCaTs. Live/dead staining shows that both RAW 264.7 (Fig. 2A) and HaCaTs (Fig. 2B) experience robust proliferation on electrospun membranes. Most cells were live (green stain) with very few dead cells (red stain) from day 1 to 7. Both cells showed a great increase in populations over time. SEM images reveal that cells are closely attached to the nanofibers. CCK-8 assays demonstrated sustained increases in cellular metabolic activity, with no discernible cytotoxic effects attributable to the electrospun nanofiber membranes (Fig. 2C and E). Moreover, both cells had viability rates greater than 90% after 24 h on electrospun membranes (Fig. 2D and F). These results suggest that the P/G-CS-OI membrane has good cytocompatibility.

Given that wound dressings are destined for direct contact with blood in vivo, assessing the potential for hemolysis is a crucial aspect of hemocompatibility testing (Fig. S2, Additional file). Results from these assays yielded supernatants that were light yellow in color post-contact with the different membranes. This outcome paralleled the negative control and stood in stark contrast to the bright red of the positive control. The P/G, P/G-CS, and P/G-CS-OI membranes were associated with hemolysis rates of less than 2%, a figure well below the international safety benchmark of 5%.

The aforementioned in vitro biocompatibility findings were validated through in vivo experiments that involved subcutaneous implantation of the electrospun nanofiber membranes in a rat model. Subsequent histological analyses at days 7 and 28 post-implantation indicated only minimal immune reactions to the P/G, P/G-CS, and P/G-CS-OI membranes, as evidenced by H&E staining (Fig. S3A, Additional file). Although encapsulated by fibroblast layers, significant infiltration by neutrophils or monocytes was notably absent. Masson’s trichrome staining illustrated tissue-related fiber proliferation and collagen deposition, indicative of integration following membrane implantation (Fig. S3B, Additional file). Measurement of the fibrous capsule thickness, one marker of immune response, revealed a decrease in thickness for samples P/G-CS and P/G-CS-OI, from 408 ± 33 μm and 237 ± 37 μm at day 7, to 233 ± 23 μm and 161 ± 14 μm at day 28, respectively (Fig. S3C, Additional file). In contrast, P/G samples exhibited an increase in capsule thickness over the same period.

Furthermore, analysis of cell infiltration depth at the tissue-membrane interface revealed lower figures for the P/G-CS and P/G-CS-OI membranes compared to P/G alone at both 7 and 28 days post-implantation (Fig. S3D, Additional file). Collectively, these findings reinforce the conclusion that the fabricated electrospun nanofiber membranes are of good biocompatibility, implying the potential for wound healing interventions.

### Macrophage anti-inflammatory modulation of P/G-CS-OI membrane

**Fig. 3:**
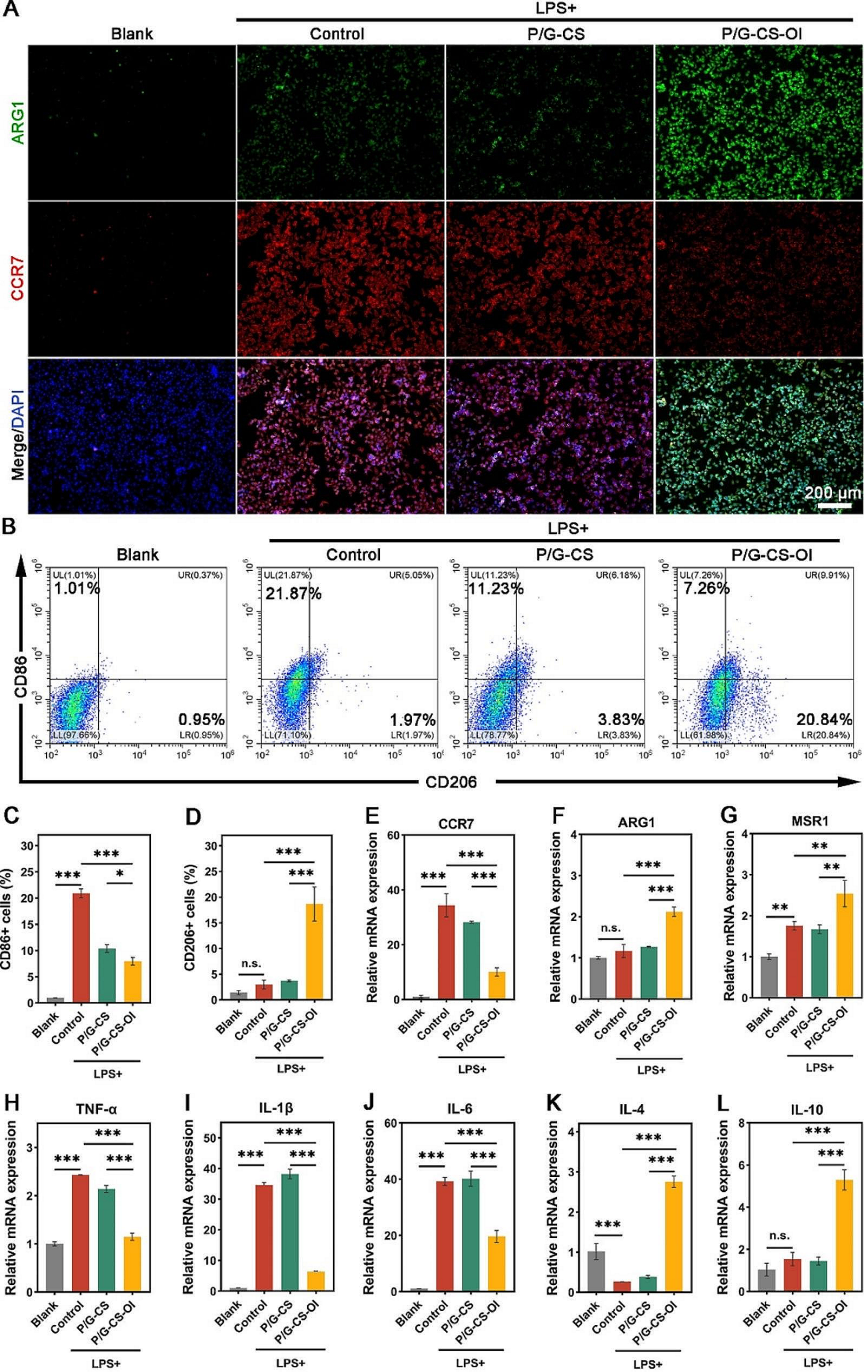
In vitro anti-inflammatory assessments. Both IF imaging (**A**) and flow cytometry (**B**-**D**) demonstrate the P/G-CS-OI membrane is capable of downregulating M1 and upregulating M2 phenotypes of macrophages. RT-PCR analysis also confirms that the P/G-CS-OI membrane downregulates M1 (**E**) and upregulates M2 (**F** and **G**) phenotypes of macrophages, as well as downregulates pro-inflammatory genes of TNF-α (**H**), IL-1β (**I**), and IL-6 (**J**) and upregulates anti-inflammatory genes of IL-4 (**K**) and IL-10 (**L**). *p < 0.05, **p < 0.01, ***p < 0.001, and n.s., not significant

Macrophages are indispensable in mounting a local immune response within injured tissues, an imbalance characterized by the predominance of M1 macrophages and impaired conversion to M2 macrophages can exacerbate tissue damage and impede wound healing processes [45]. Therefore, we investigated the impact of the P/G-CS-OI membranes on macrophage polarization under LPS challenge (Fig. 3). Figure 3A shows a notable upregulation of the levels of CC Chemokine Receptor 7 (CCR7) upon LPS exposure. Remarkably, the application of P/G-CS-OI membranes led to significant attenuation in the fluorescence signal of CCR7, concomitantly with an upsurge in the Arginase 1 (ARG1) signal, indicative of a shift towards M2 macrophage polarization. Flow cytometry analysis reveals a substantial decrease (7.26%) in the percentage of M1 macrophages (CD86+), alongside a marked increase (20.84%) in M2 macrophages (CD206+), after P/G-CS-OI treatment (Fig. 3B-D).

To further elucidate the role of P/G-CS-OI in macrophage polarization and the regulation of inflammatory mediators, RT-PCR was employed (Fig. 3E-L). The RT-PCR results demonstrated a significant increase in the gene expression of CCR7, TNF-α, IL-1β, and IL-6 in LPS-activated RAW264.7 cells, coupled with a marked decrease in IL-4 expression. Interestingly, the level of IL-10 did not exhibit the anticipated decrease, suggesting an inherent cellular self-regulation mechanism aimed at preventing excessive inflammation. In the P/G-CS-OI group, RT-PCR results indicated a substantial downregulation in the expression of pro-inflammatory genes (CCR7, TNF-α, IL-1β, and IL-6) and upregulation in ARG1, Macrophage Scavenger Receptor 1 (MSR1), IL-4, and IL-10, underscoring the potent immunomodulatory and pro-repair capabilities of the P/G-CS-OI membranes. It is noteworthy that MSR1, as one of the markers of M2 macrophages, can enhance the phagocytosis ability of macrophages. This facilitates the clearance of bacterial fragments and apoptotic cells, thereby contributing to the improvement of the chronic wound inflammatory microenvironment [46].

Macrophage polarization refers to the activation state of macrophages in a given time and space within an inflammatory/homeostatic environment, characterized by sufficient plasticity and flexibility to integrate the influences of various signals from the surroundings, providing a general outline of the tissue state, without granularity. The polarization state of macrophages can be determined via specific cytokines and molecular markers [47]. Previous experiments have demonstrated that OI can effectively activate the Nuclear Factor Erythroid 2-Related Factor 2 (NRF2), preventing M1 macrophage polarization by inhibiting NF-κB nuclear translocation. Additionally, the polarization of macrophages was shifted to M2 phenotype through activation of the PPARγ signaling pathway and autophagy [48, 49]. Our findings further demonstrated that the use of P/G-CS-OI membranes, not only promotes the transformation of macrophages from the M1 to the M2 phenotype but also enhances their phagocytic function and anti-inflammatory activity. This dual action considerably augments the potential for improved skin repair in chronic wound scenarios, highlighting the therapeutic value of P/G-CS-OI membranes in modulating the immune response and facilitating tissue regeneration.

### Antioxidant activity of P/G-CS-OI membrane

**Fig. 4:**
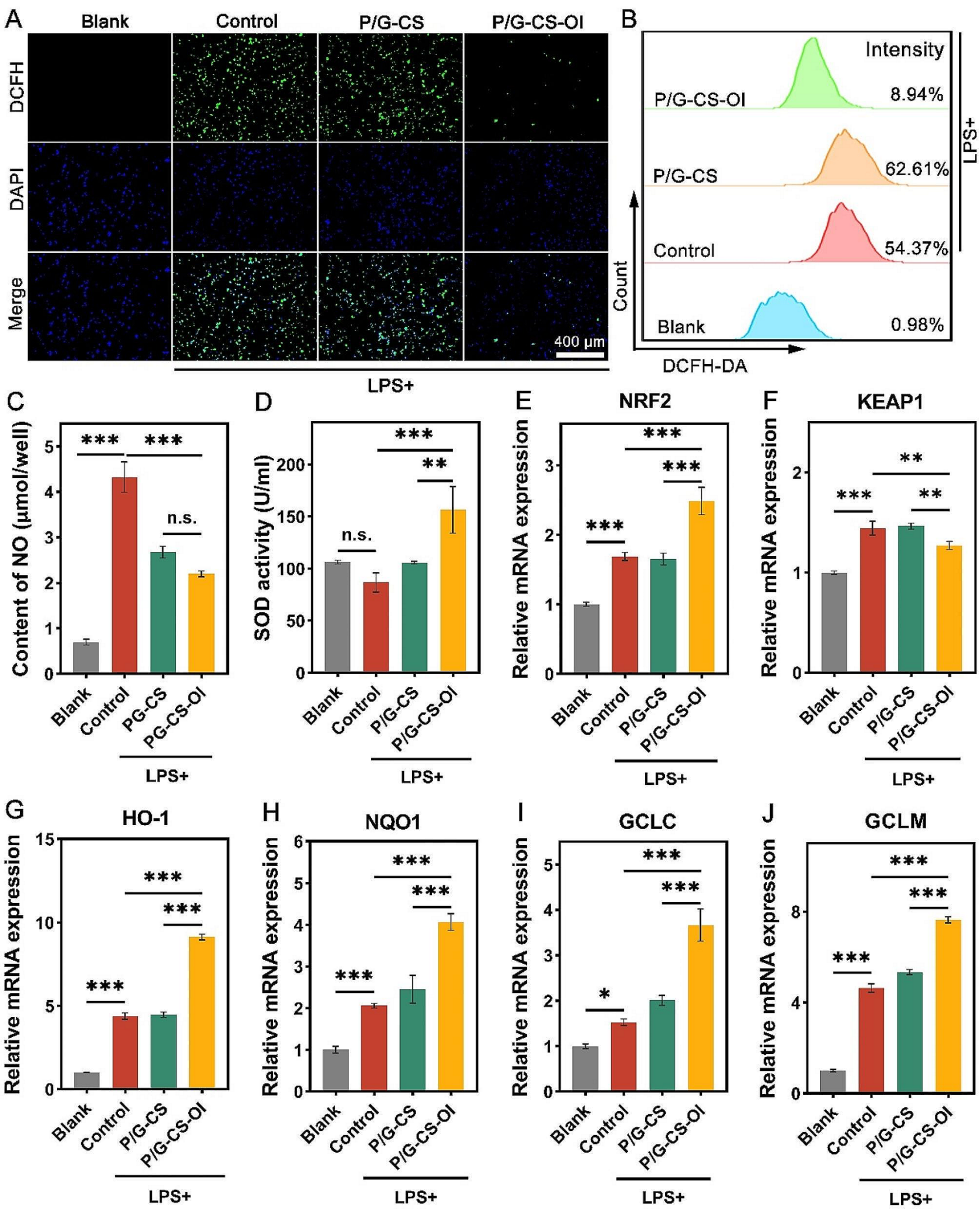
In vitro antioxidative assessments. DCFH-DA staining (**A**) and flow cytometry (**B**) analysis reveal that the P/G-CS-OI membrane shows the greatest ROS clearance of LPS-challenged macrophages. It also significantly lowers the NO production (**C**) and enhances SOD activity (**D**) of LPS-challenged macrophages. RT-PCR analysis demonstrates that the P/G-CS-OI membrane upregulates NRF2 (**E**) and downregulates KEAP1 (**F**), as well as upregulates NRF2 downstream genes of HO-1 (**G**), NQO1 (**H**), GCLC (**I**), and GCLM (**J**).*p < 0.05, **p < 0.01, ***p < 0.001, and n.s., not significant

The excessive ROS accumulation in diabetic wounds can not only oxidize surrounding lipids and proteins, causing cellular damage but also promote the chemotaxis of inflammatory factors to the wound site, thus hindering skin regeneration [50]. Therefore, the development of wound dressings with antioxidant capabilities has become the focus of current research [51]. To this end, RAW 264.7 cells were cultured in a pathological oxidative microenvironment, induced by 100 ng/mL LPS, and intracellular ROS levels were assessed using DCFH-DA. The resulting fluorescence signal was quantified and characterized using both flow cytometry and fluorescence microscopy. The results, as illustrated in Fig. 4A, indicate that in the LPS-treated groups (Control and P/G-CS), DCFH-DA exhibited significantly higher green fluorescence compared to the control group. Notably, the use of P/G-CS-OI membranes effectively reduced intracellular oxidative stress. Flow cytometry quantification shows that the percentage of FITC-positive channels in the P/G-CS-OI group was significantly lower compared to other experimental groups (Fig. 4B). This finding highlights the potent ROS scavenging ability of P/G-CS-OI membranes, underscoring its potential as an effective antioxidant in wound dressing applications.

Nitric Oxide (NO) is a versatile signaling molecule produced by various cell types and plays a pivotal role in inflammation and oxidative stress in skin tissue [52]. Being a free radical, NO can react with superoxide anions to form peroxynitrite, a potent oxidizing agent that inflicts damage on surrounding cells and tissues [53]. Superoxide Dismutase (SOD) is a metalloenzyme that catalyzes the disproportionation of superoxide anions, thereby maintaining cellular REDOX balance [54]. Our study demonstrates that the application of P/G-CS and P/G-CS-OI membranes significantly reduces NO levels, increases SOD activity, and consequently mitigates cellular oxidative stress damage, as shown in Fig. 4C and D.

In chronic diabetic wounds, persistent hyperglycemia induces an accumulation of damaged mitochondria, leading to an overproduction of coenzyme Q and subsequent impairment of the normal electron transport within the respiratory chain [55]. This mitochondrial dysfunction redirects a substantial number of electrons back to mitochondrial complex I, creating a significant imbalance between ROS production and elimination [56]. Such an imbalance can result in lipid peroxidation within macrophages, leading to stromal swelling and rupture of the outer membrane [57]. NRF2 is a key transcription factor that coordinates cellular defense mechanisms against oxidative and electrophilic stress [58]. It regulates the transcription of antioxidant genes dependent on the Antioxidant Response Element (ARE). This regulation includes the expression of genes such as NAD(P)H Quinone Dehydrogenase 1 (NQO1), Glutamate Cysteine Ligase Catalytic Subunit (GCLC), Glutamate Cysteine Ligase Modifier Subunit (GCLM), and Heme Oxygenase 1 (HO-1) [59]. Kelch-like ECH-associated protein 1 (KEAP1), a dimeric protein, normally binds to NRF2 in the cytoplasm and facilitates its degradation via the proteasome system. Hence, KEAP1 is also recognized as an inhibitor of NRF2. Our results confirmed that P/G-CS-OI membranes could significantly up-regulate the expression of NRF2 and its downstream antioxidant components NQO1, GCLC, GCLM, and HO-1 (Fig. 4E-J), thereby clearing excess ROS and maintaining REDOX homeostasis in the cellular environment. This result is mainly attributable to OI, which plays a crucial role in this regulatory mechanism by alkylating cysteine residues (151, 257, 288, 273, and 297) on KEAP1. This modification prompts the dissociation of NRF2 from KEAP1, allowing NRF2 to translocate to the nucleus where it interacts with ARE, thereby enhancing the expression of downstream genes with antioxidant properties and creating suitable conditions for the healing of chronic wounds. However, since the main function of OI is to alkalize KEAP1 and promote its dissociation, the influence on the expression level of KEAP1 is relatively small (Fig. 4F) [60].

### Bioactivity of P/G-CS-OI for proliferation of keratinocytes

**Fig. 5:**
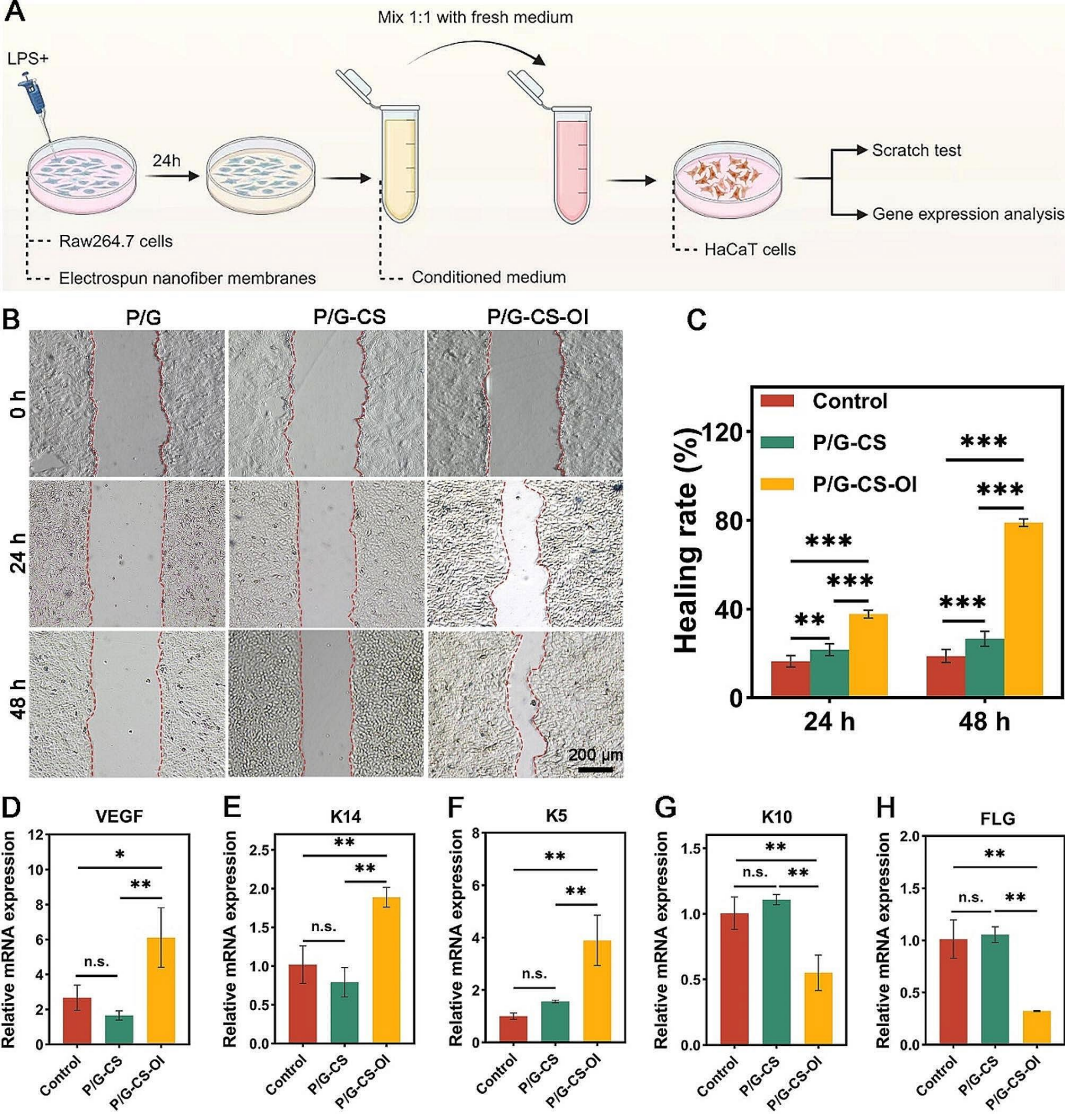
Effects of P/G-CS-OI membrane treated macrophage conditioned medium on HaCaT migration and gene expression. Schematic of experimental design (**A**). Conditioned medium significantly promotes HaCaT migration (**B**) as evidenced by accelerated healing rate of scratch (**C**). It significantly upregulates VEGF expression (**D**) and upregulates K14 (**E**) and K5 (**F**) expressions and downregulates K10 (**G**) and FLG (**H**) expressions. *p < 0.05, **p < 0.01, ***p < 0.001, and n.s., not significant

To evaluate the effect of macrophage immune response on the formation and differentiation of blood vessels and epidermis, RAW 264.7 cells were planted in different groups of electrospun nanofiber membranes and activated with LPS to simulate the inflammatory microenvironment. The conditioned medium obtained was used to grow HaCaTs and evaluate the effects of cytokines and growth factors produced by macrophages under different conditions on the differentiation and maturation of HaCaTs (Fig. 5A).

We investigated the effects of different medium conditions on HaCaT migration through scratch experiments. As shown in Fig. 5B, neither P/G nor P/G-CS showed enhanced cell migration, while P/G-CS-OI showed a higher ability to promote cell migration. This trend was consistent with the polarization change of macrophages, indicating that the conditioned medium obtained by macrophages after remodeling had obvious chemotactic effect on epidermal repair cells. However, the mechanism underlying accelerated HaCaT migration remains unknown in the current study.

We further measured gene expression in different groups of RAW 264.7 macrophages and HaCaTs. We found that RAW 264.7 cells up-regulated VEGF under the influence of P/G-CS-OI membranes (Fig. 5D), VEGF can promote endothelial cell migration, proliferation, and differentiation. It also helps to form new blood vessel structures, providing enough oxygen and nutrients to promote growth, differentiation, and recovery of damaged tissues. Based on good vascularization, the proliferation and differentiation of keratin can provide an important structural role in wound healing. Keratin 14 (K14), a member of the type I keratin family, is always paired with type II Keratin 5 (K5), and both are predominantly expressed in the undifferentiated basal cell layer of skin. These keratins serve as the foundational components for the assembly of lamellar squamous epithelial keratins, forming a robust cytoskeletal network that could counteract the mechanical stress and cellular instability typically observed in diabetic wounds [61]. In addition, keratin 10 (K10) and filaggrin (FLG) are closely associated with terminal differentiation in the epidermis of the skin [62]. The increased ratio of K14/K10 often indicates that epidermal cells are in a period of rapid proliferation [63]. We observed significantly upregulated expression of K14 (Fig. 5E) and K5 (Fig. 5F) as well as significantly down-regulated expression of K10 (Fig. 5G) and FLG (Fig. 5H) of HaCaTs for the P/G-CS-OI group. These results indicate that P/G-CS-OI membrane-primed macrophages might promote the proliferation of keratinocytes via a paracrine manner. While it is unclear if the P/G-CS-OI membrane has a direct effect on HaCaT proliferation.

### Diabetic wound healing

**Fig. 6:**
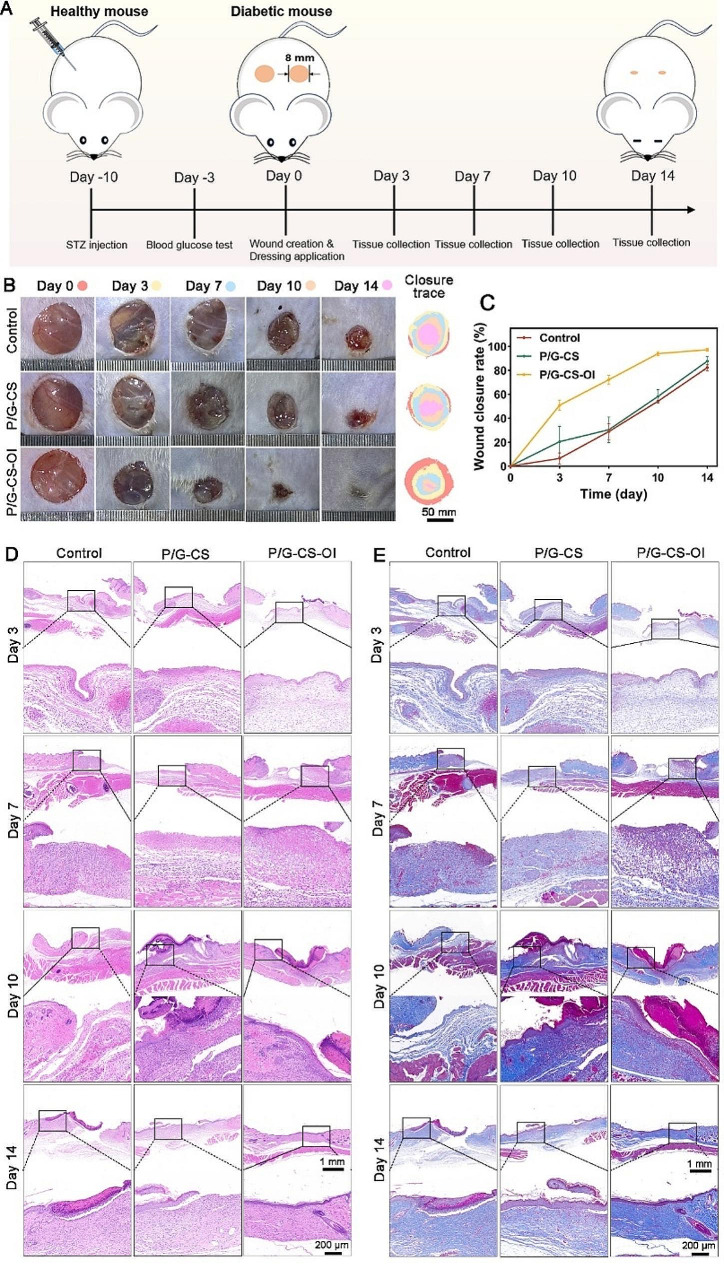
Gross observation and histological analyses of wound healing quality. Schematic of diabetic mouse preparation and wound dressing treatment with subsequent analysis endpoint (**A**). Gross images of wounds (**B**) with plotted wound closure curves (**C**). Histological images of H&E (**D**) and Masson’s trichrome (**E**) staining from day 3 to 14

In vivo studies were conducted to assess the capacity of the P/G-CS-OI membrane in promoting diabetic full-thickness skin wound healing in an STZ-induced diabetic mouse model. Wound healing process was observed at days 0, 3, 7, 10, and 14 (Fig. 6A). As shown in Fig. 6B and C, the P/G-CS-OI membranes already showed a higher wound closure rate (50 ± 4%) compared with P/G (7 ± 5%) and P/G-Cs (20 ± 13%) at day 3. After 14 days, P/G-CS-OI membrane-treated wounds were almost completely healed, while wounds of the other groups showed delayed wound repair.

To further elucidate the role of electrospun nanofiber membranes in promoting chronic wound healing, histological analyses were performed (Fig. 6D and E). H&E staining images show that compared with P/G and P/G-CS groups, there was more neovasculation and hair follicle formation in P/G-CS-OI membrane-treated wounds with less inflammatory cell infiltration. After 14 days, complete re-epithelialization was observed in the P/G-CS-OI membrane-treated wounds, characterized by typical cortical structures and morphological features (Fig. 6D). Additionally, the thickness of the granulation tissue, a vital indicator of the wound healing process, was assessed using Masson trichrome staining to determine collagen deposition and scar length in the tissue. Compared with P/G and P/G-CS treated wounds, the granulation tissue thickness of the P/G-CS-OI membrane-treated wound is thicker with significantly reduced wound margin. Dense and orderly collagen accumulation under the epidermis was observed in the P/G-CS-OI membrane-treated wound, indicating mature regenerated skin tissue (Fig. 6E). In contrast, the other groups showed inferior skin regeneration as evidenced by sparse collagen deposition and remaining wound margin.

**Fig. 7:**
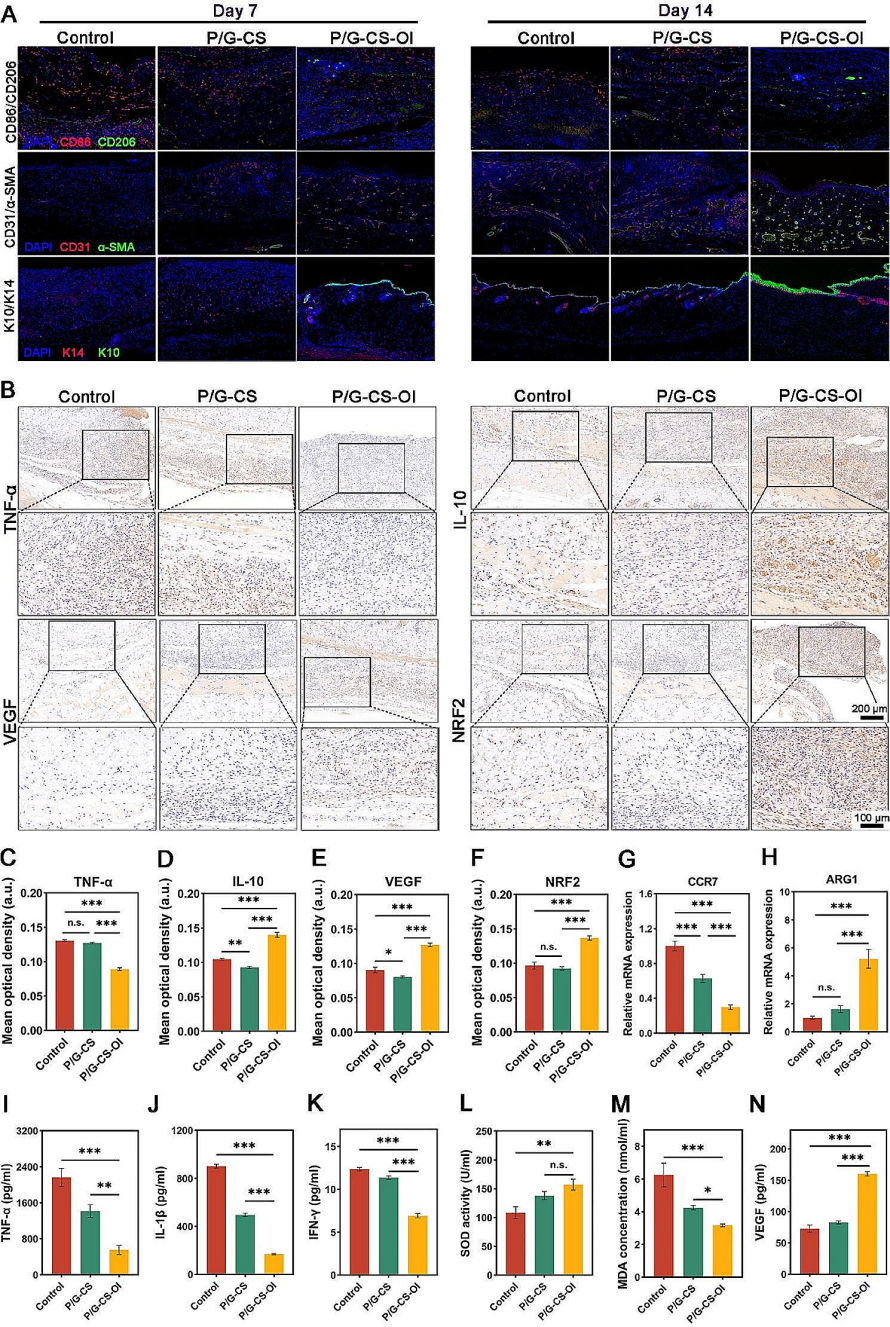
IF and IHC staining and ELISA analyses of wound healing quality. IF staining shows that the P/G-CS-OI membrane modulates macrophage polarization by down-regulating CD86 + cells and up-regulating CD206 + cells (**A**). The P/G-CS-OI membrane improves neovascularization as evidenced by increased numbers CD31+/α-SMA + blood vessels and promotes epithelialization by intense K10+/K14 + layers (**A**). IHC staining (**B**) shows that the P/G-CS-OI membrane significantly alleviates inflammation by down-regulating TNF-α expression (**C**) and upregulating the expressions of IL-10 (**D**), VEGF (**E**), and NRF2 (**F**). RT-PCR analysis of healing tissues also confirms that the **P**/G-CS-OI membrane downregulates M1 (**G**) and upregulates M2 (**H**). ELISA analysis of healing tissues also confirms the P/G-CS-OI membrane significantly reduces the pro-inflammatory cytokine accumulation of TNF-α (**I**), IL-1β (**J**), and IFN-γ (**K**). It shows powerful in vivo antioxidative capacity by stimulating SOD (**L**) and reducing MDA (**M**) production and also upregulating VEGF secretion (**N**).*p < 0.05, **p < 0.01, ***p < 0.001, and n.s., not significant

Chronic wounds are hallmarked by prolonged inflammation, which is characterized by a dominant M1 macrophage polarization, elevated levels of inflammatory cytokines, impaired angiogenesis and epithelial regeneration [64]. In addition to in vitro results of P/G-CS-OI membranes facilitating macrophage phenotypic remodeling, our study further investigated the modulatory effects of the P/G-CS-OI membrane on macrophages in a diabetic mouse wound model. Immunofluorescence images (Fig. 7A) with fluorescence intensity quantification (Fig. S4A and 4B, Additional file) show that the P/G-CS-OI membrane-treated wound presented significantly greater populations of M2 macrophage (CD206+) and smaller populations of M1 macrophage (CD86+) within the healing tissues compared with the P/G-CS membrane-treated and control groups at days 7 and 14. Gene expression is one of the simplest ways to identify the polarization state of macrophages [65]. We further confirmed a higher gene expression of ARG1 (M2 phenotype) and a lower gene expression of CCR7 (M1 phenotype) for the P/G-CS-OI membrane-treated wound in comparison to the other groups (Fig. 7G and H). Taken together, these results indicate that the P/G-CS-OI membrane effectively promotes M1/M2 transition in diabetic wounds. Recently, we and other groups have proposed many other approaches for treating diabetic wounds and observed similar findings [66–68]. Inspired by these positive results, future work should focus on establishing quantifiable criteria and indexes for the polarization shift from M1 to M2 in a temporospatial manner to advance these exciting findings to pre-clinical investigations, even going further. These proposed therapies should be compared with M1/M2 transitions in wounds of healthy individuals and diabetic subjects to determine critical checkpoints and quantifiable criteria [69]. For each proposed therapy itself, comprehensive investigations of potential immunomodulatory pathways at cellular and molecular levels should be performed.

To further confirm the promotive effects of the P/G-CS-OI membrane on diabetic wound healing, we performed immunofluorescence and immunohistochemical staining to evaluate the quality of regenerated tissues (Fig. 7A and B). Impaired angiogenesis is a hallmark of diabetic wounds. To quantitatively analyze neovascularization within the granulation tissue, immunofluorescence staining for CD31 and α-Smooth Muscle Actin (α-SMA) was employed, which showed that the P/G-CS-OI membrane significantly upregulated the expression of both CD31 and α-SMA in the regenerated skin on days 7 and 14 (Fig. 7A; Figs. S4C and 4D, Additional file). CD31 can label endothelial cells responsible for the formation of vascular lumen, and α-SMA can label pericytes/smooth muscle cells involved in structural stability and regulation of vascular permeability. Vessels displaying both CD31 + and α-SMA + were characterized as mature, whereas vessels labeled only with CD31 + were considered immature [70]. Our findings suggest that electrospun nanofiber membranes are effective in promoting the formation of mature blood vessels.

K10 and K14 serve as critical markers for epidermal differentiation and epidermal basal cells, respectively [71]. Fluorescence results showed that P/G and P/G-CS groups had weak K10 + epidermis and scattered K14 + epidermal basal layer, indicating that the wound tissue had not regenerated a mature epidermal structure (Fig. 7A; Figs. S4E and 4F, Additional file). In contrast, wounds treated with P/G-CS-OI membranes displayed a well-formed epidermis, characterized by a completely covered K10 + epidermal basal layer and a K14 + epidermal lining. This finding demonstrates that P/G-CS-OI membranes promote rapid and effective epithelialization of wounds.

After 7 days of treatment under the same conditions, immunohistochemical staining showed the expression of TNF-𝛼 in the P/G-CS-OI group was significantly decreased, while the expression of IL-10, VEGF, and NRF2 was significantly increased (Fig. 7B-F), which was aligned with the in vitro results. ELISA was also used to quantitatively analyze the expressions of TNF-𝛼, IL-1β, Interferon (IFN)-γ, and VEGF, and corresponding kits were used to detect the expressions of oxidase SOD and oxidative stress product malondialdehyde (MDA) (Fig. 7I-N). The inflammatory cytokines TNF-𝛼, IL-1β, and IFN-γ inhibit wound healing, while VEGF acts on endothelial cells to aid endothelial chemotactic migration, which is essential for angiogenesis during cell proliferation. These outcomes are consistent with observations from immunohistochemistry and immunofluorescence staining, as anticipated. The expressions of TNF-𝛼, IL-1β, IFN-γ, and MDA were significantly decreased, while the expressions of VEGF and SOD were increased. These results suggest that the P/G-CS-OI membrane not only inhibits tissue inflammation but also induces regeneration of blood vessels and skin appendages and promotes wound healing.

Given the great modulatory effects of the P/G-CS-OI nanofiber membrane on macrophages, we herein envision that it might show promise in other biomedical fields, where macrophage-modulating effects are indispensable. These include but are not limited to anti-scarring dressings, post-surgery antiadhesion membranes, and drug delivery carrier implants. However, it should be noted that some limitations exist in the current study. First, we used a two-step method for CS-OI coating onto nanofibers, during which nanofibers were immersed in 1 mg/mL CS-OI solution and crosslinked by EDC/NHS. Although our results show that the resulting nanofiber membranes have great modulatory effects on macrophages both in in vitro and in vivo studies and show no significant toxic effects in subcutaneous implantation, future studies should be taken to optimize the concentrations of coated CS-OI and to comprehensively investigate its potential side effects in vivo. Secondly, diabetic wounds present a complex inflammatory environment. Although STZ-induced rodents have been extensively used in wound healing assessment, their pathology might be distinct from that of human DMs. In addition, the skin histology and healing processes of small animals may not sufficiently reflect that of human skin. Future studies should consider diabetic models of large animals such as dogs and pigs that are more relevant to human DMs. Last, we have primarily targeted the modulatory effects of OI on macrophages in the current study, more intercellular interactions such as those involving neutrophils and dendritic cells should be considered in future studies [72].

## Conclusions

In summary, our study focused on the development and characterization of a bioactive electrospun nanofiber membrane, composed of PCL, Gelatin, CS, and OI, envisioned as an innovative wound dressing material. The P/G-CS-OI membranes demonstrate a remarkable ability to inhibit the activation of specific inflammatory mediators, effectively contributing to macrophage modulation, and thereby promoting the healing of chronic wounds. A noteworthy aspect of the P/G-CS-OI membrane is its capacity for the sustained release of OI. This feature is instrumental in activating ARE through the NRF2/KEAP1 pathway, which relieves oxidative stress and promotes wound healing. In vivo studies in a diabetic mouse skin wound model further validated the significant therapeutic benefits of the P/G-CS-OI membrane. These benefits include inflammatory modulation, promoted neovascularization, and enhanced collagen deposition, though it remains a challenge to extrapolate data from STZ-diabetic mouse models to those of humans. Therefore, this inherently multifunctional electrospun nanofiber membrane not only reconstructs the tissue microenvironment but also accelerates tissue regeneration. Its diverse and potent capabilities position it as a promising biological dressing, meriting further exploration and potential applications in tissue engineering and regenerative medicine.

### Electronic supplementary material

Below is the link to the electronic supplementary material.

Supplementary Material 1

## Data Availability

No datasets were generated or analysed during the current study.

## Abbreviations

α-SMA: α-Smooth muscle actin
DAPI: 4’,6-diamidino-2-phenylindole
DCFH-DA: 2,7dichlorodihydrofluorescein diacetate
ELISA: Enzyme-linked immunosorbent assay
FITC: Fluorescein isothiocyanate
FTIR: Fourier transform infrared reflection
HO-1: Heme Oxygenase-1
IFN: Interferon
IHC: Immunohistochemical
IL: Interleukin
KEAP1: Kelch-like ECH-associated protein 1
LPS: Lipopolysaccharide
MDA: Malondialdehyde
NQO1: NAD(P)H:quinone oxidoreductase 1
NRF2: Nuclear factor (erythroid derived 2)-related factor 2
OI: 4-octyl itaconate
PBS: Phosphate Buffer Saline
PCL: Polycaprolactone
ROS: Reactive oxygen species
RT-PCR: Real‑time polymerase chain reaction
SEM: Scanning electron microscopy
SOD: Superoxide Dismutase
STZ: Streptozocin
TAC: Tricarboxylic acid cycle
TNF: Tumor necrosis factor
XRD: X-ray Diffraction
