# Supplementary material for: Anti-inflammatory and anti-oxidative electrospun nanofiber membrane promotes diabetic wound healing via macrophage modulation
Source: J Nanobiotechnology. 2024 Mar 16;22:116. doi: 10.1186/s12951-024-02385-9 (PMC10943854; doi:10.1186/s12951-024-02385-9)
Supplement: Supplementary file 1 — Supplementary Material 1 [file 12951_2024_2385_MOESM1_ESM.docx]

**Additional file**

**Table S1.** Quantitative Real-time PCR primer sequences

| Gene | Forward | Reverse |
| --- | --- | --- |
| TNF-α | CAGGCGGTGCCTATGTCTC | CGATCACCCCGAAGTTCAGTAG |
| IL-4 | GGTCTCAACCCCCAGCTAGT | GCCGATGATCTCTCTCAAGTGAT |
| IL-6 | CTGCAAGAGACTTCCATCCAG | AGTGGTATAGACAGGTCTGTTGG |
| IL-10 | CTTACTGACTGGCATGAGGATCA | GCAGCTCTAGGAGCATGTGG |
| CCR7 | CAGGTGTGCTTCTGCCAAGAT | GGTAGGTATCCGTCATGGTCT |
| ARG1 | CTCCAAGCCAAAGTCCTTAGAG | GGAGCTGTCATTAGGGACATCA |
| NRF2 | TCTTGGAGTAAGTCGAGAAGTGT | GTTGAAACTGAGCGAAAAAGGC |
| KEAP1 | TGCCCCTGTGGTCAAAGTG | GGTTCGGTTACCGTCCTGC |
| HO-1 | AAGCCGAGAATGCTGAGTTCA | GCCGTGTAGATATGGTACAAGGA |
| GCLC | CTACCACGCAGTCAAGGACC | CCTCCATTCAGTAACAACTGGAC |
| GCLM | AGGAGCTTCGGGACTGTATCC | GGAAACTCCCTGACTAAATCGG |
| MSR1 | TGGAGGAGAGAATCGAAAGCA | CTGGACTGACGAAATCAAGGAA |
| K10 | GCCTCCTACATGGACAAAGTC | GCTTCTCGTACCACTCCTTGA |
| K14  GAPDH | AGCGGCAAGAGTGAGATTTCT  AGGTCGGTGTGAACGGATTTG | CCTCCAGGTTATTCTCCAGGG  GGGGTCGTTGATGGCAACA |


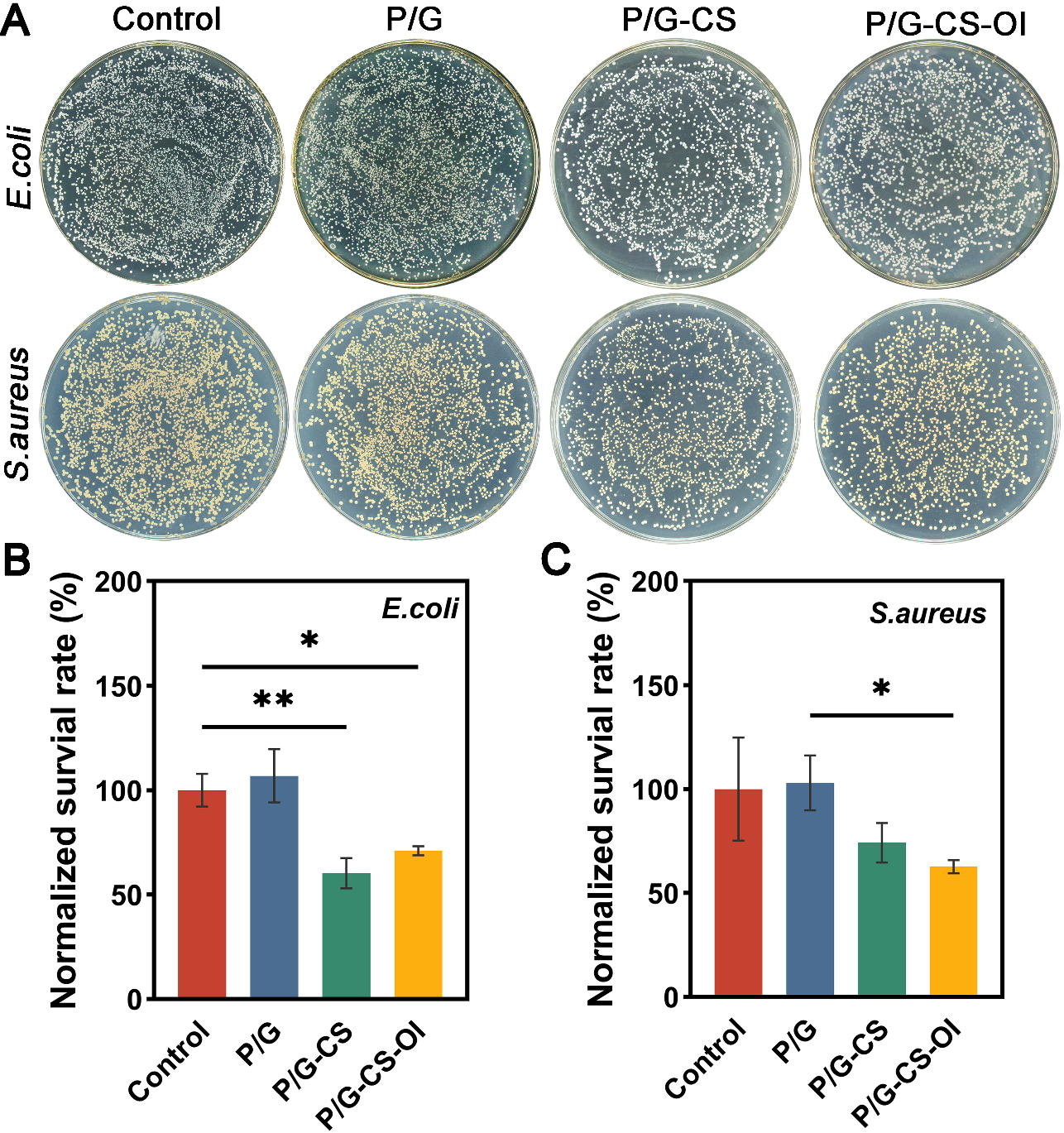


**Fig. S1.** *In vitro* antibacterial properties of electrospun nanofiber membranes. The P/G-CS-OI membrane significantly inhibited the growth of *E. coli* and *S. aureus* (A) by quantifying the normalized survival of *E. coli* (B) *and S. aureus* (C). **p* < 0.05, ***p* < 0.01.


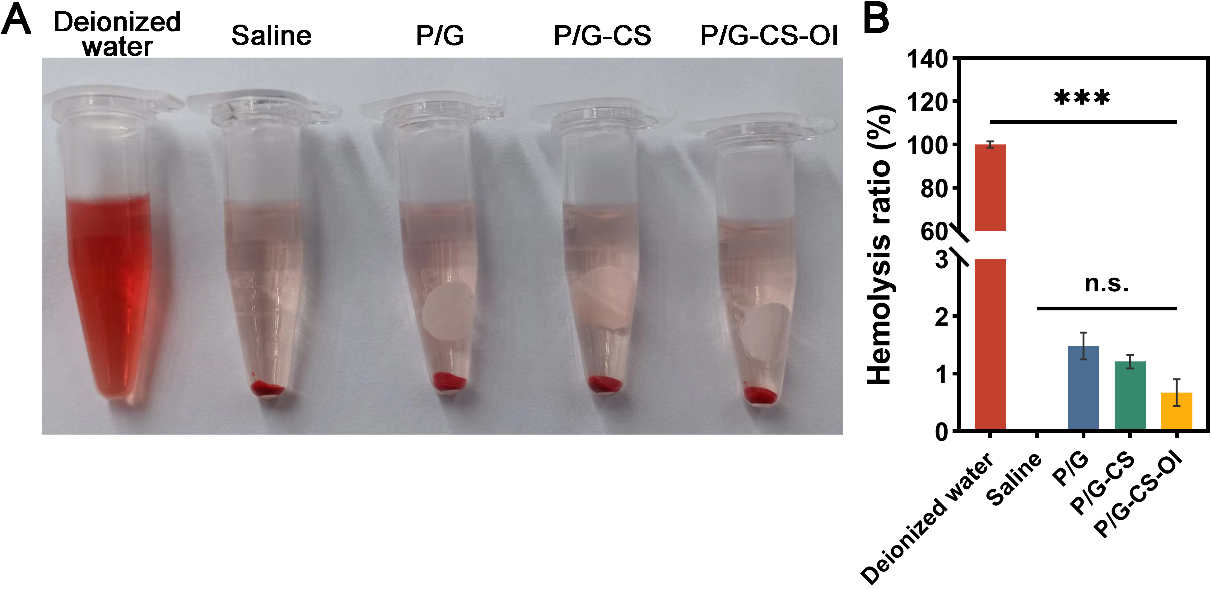


**Fig. S2.** Hemocompatibility of electrospun nanofiber membranes. Photos of the hemolysis assay (A) and hemolysis rate (B) of electrospun nanofiber membranes. ****p* < 0.001, and n.s., not significant.


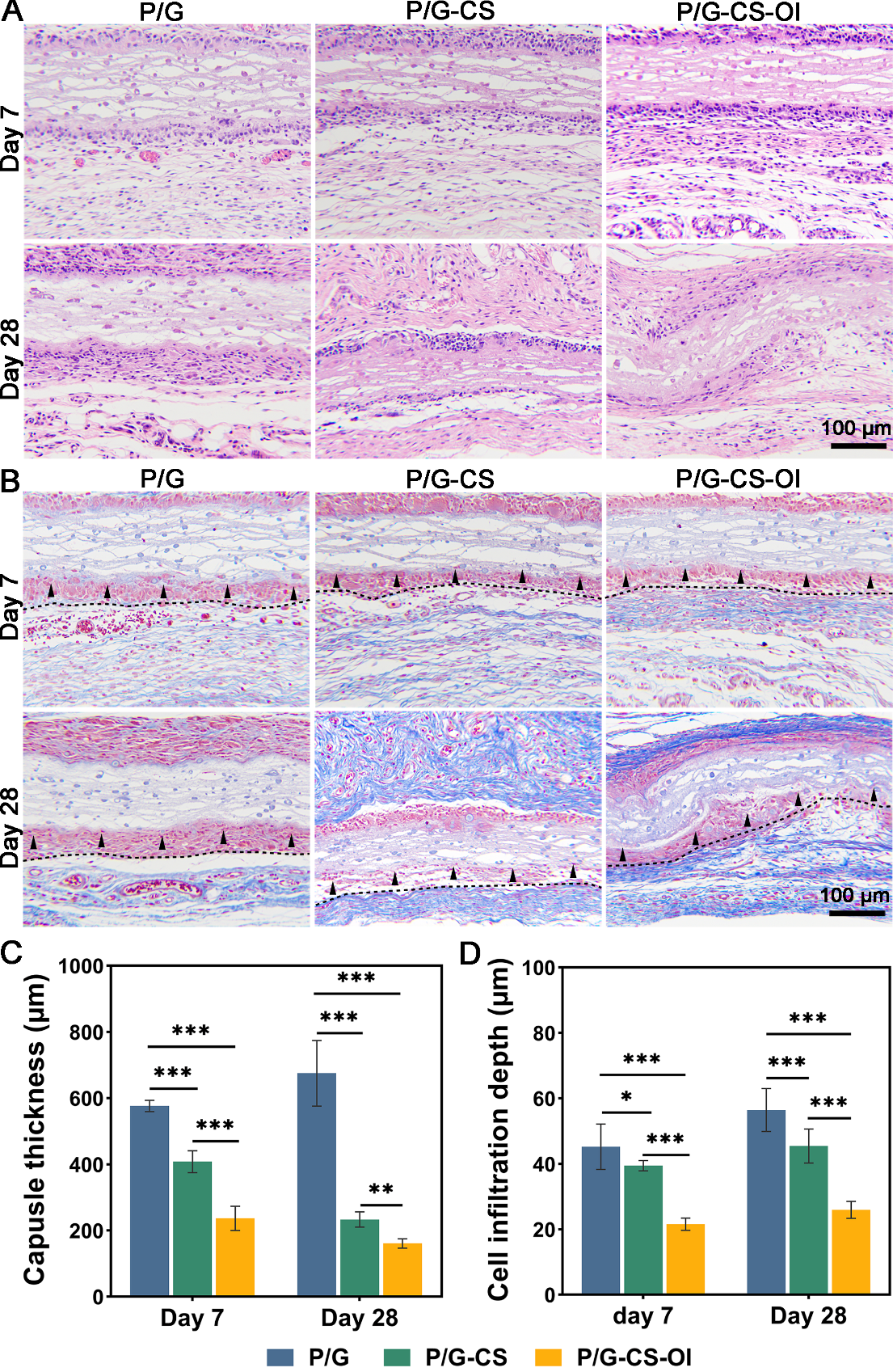


**Fig. S3.** Histological analyses of subcutaneous embedding. Histological images of H&E (A) and Masson’s trichrome (B) staining from day 7 to 28 with quantification of capsule thickness (C) and cell infiltration depth (D) **p* < 0.05, ***p* < 0.01, ****p* < 0.001.


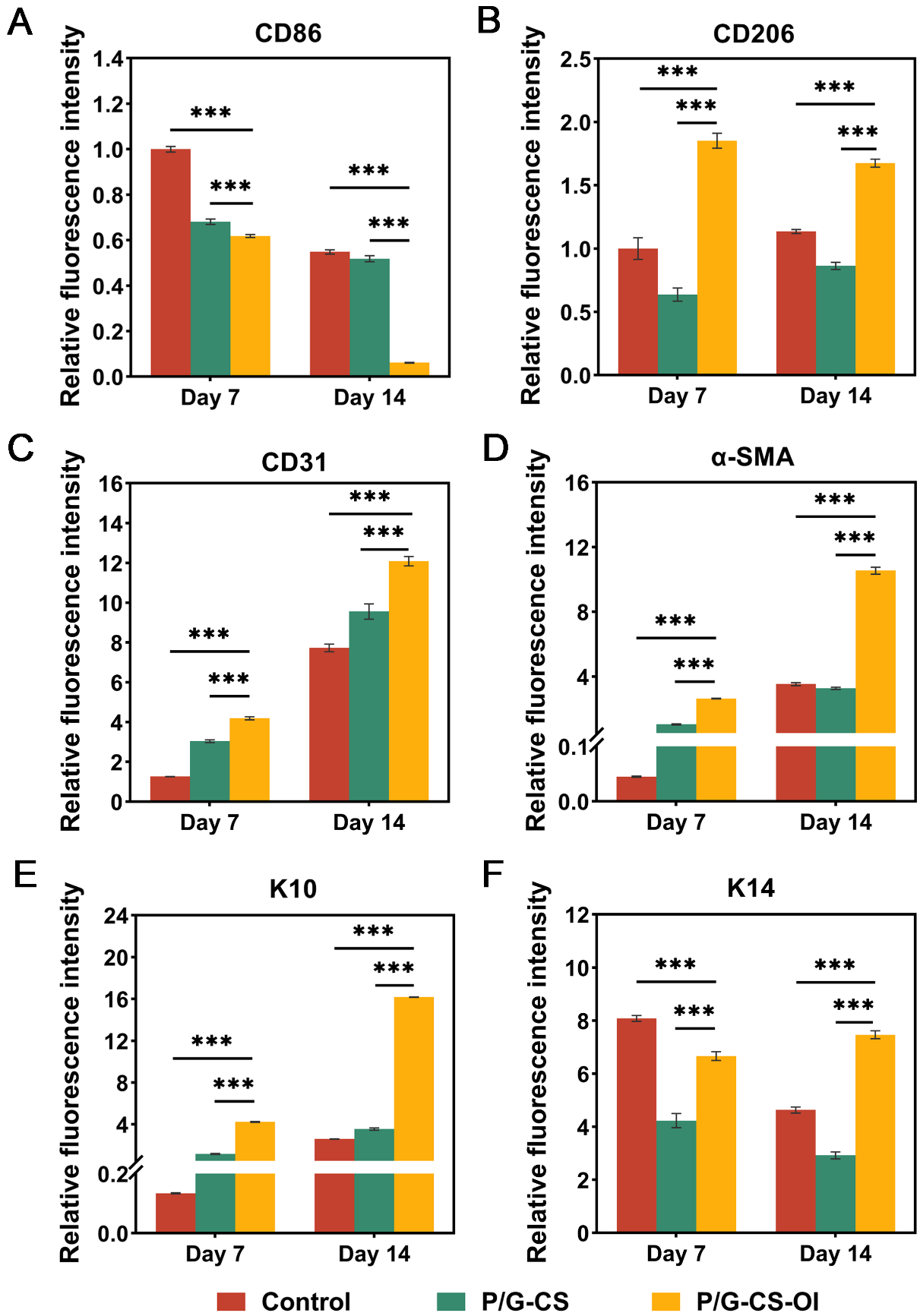


**Fig. S4.** The quantification procedure for IF images of wound healing quality. The quantification of IF staining shows that P/G-CS-OI membrane significantly alleviates the inflammatory response by reducing CD86+ cells (A) and increasing CD206+ cells (B). P/G-CS-OI membrane promotes angiogenesis by increasing the number of CD31+/α-SMA+ vessels (C and D) and strong K10+/K14+ layer (E and F) to promotes epithelialization. ****p* < 0.001.
